# Effects of exercise intensity and volume on systemic inflammation in overweight and obese postmenopausal women: a dose-response meta-analysis

**DOI:** 10.3389/fimmu.2026.1801309

**Published:** 2026-03-19

**Authors:** Gang Huang, Guanbo Wang, Lianghao Zhu, Ping Wang

**Affiliations:** 1 School of Physical Education, Hunan University of Science and Technology, Xiangtan, China; 2 Physical Education Institute, Sichuan University of Science & Engineering, Zigong, China; 3 Key Laboratory of Psychological and Physiological Regulation in Competitive Sports, Tianjin University of Sport, Tianjin, China; 4 School of Physical Education, Guangdong University of Petrochemical Technology, Maoming, China

**Keywords:** %HRmax, dose-response, exercise, inflammation, menopause, meta-regression, obesity

## Abstract

**Objective:**

This study evaluates the dose-response relationships between multiple exercise parameters (volume, duration, and intensity) and inflammatory modulation (C-reactive protein [CRP], interleukin-6 [IL-6], tumor necrosis factor-α [TNF-α], and adiponectin) exclusively in overweight and obese postmenopausal women.

**Methods:**

Five databases were systematically searched for randomized controlled trials (RCTs) up to January 2026 (PROSPERO: CRD420261288134). Standardized Mean Differences (SMD) were estimated using random-effects models. Meta-regression analyses investigated the dose-response effects of total exercise volume (MET-minutes/week), intervention duration (weeks), and exercise intensity (systematically standardized to %HRmax).

**Results:**

Analysis of 30 RCTs (N = 2,124) demonstrated that exercise interventions significantly reduced TNF-α (SMD = -0.47, p < 0.001) and CRP (SMD = -0.36, p = 0.001). Changes in IL-6 and adiponectin were not statistically significant. Leave-one-out sensitivity analyses confirmed the exceptional stability of the CRP (SMD range: -0.41 to -0.28) and TNF-α reductions. Meta-regression revealed no definitive linear dose-response relationships for total exercise volume or duration across any biomarkers. However, a borderline trend (p = 0.063) indicated that higher exercise intensity is associated with greater reductions in TNF-α.

**Conclusion:**

Structured exercise functions as a potent therapeutic modality that reliably mitigates systemic and tissue-derived inflammation in postmenopausal obesity. The findings challenge the traditional reliance on total exercise volume or duration, pointing instead toward an “intensity threshold” where higher exercise intensities more effectively drive anti-inflammatory adaptations. Precision exercise prescriptions for this demographic should prioritize the optimization of exercise intensity.

## 1 Introduction

Overweight and obesity are no longer defined simply as disorders of positive energy balance but are recognized globally as complex pathological states (1). A hallmark of this condition is chronic, low-grade systemic inflammation (CLGSI), often termed “meta-inflammation,” which serves as the key pathophysiological basis for insulin resistance and cardiovascular disease (CVD) (2, 3). For women, menopause represents a critical biological turning point that often constitutes a “second hit” to metabolic health (4). With the decline in ovarian function and the withdrawal of endogenous estrogens, body fat distribution shifts significantly from subcutaneous to visceral adipose tissue (VAT) (5). In the resulting “menopause-obesity-CLGSI” vicious cycle, postmenopausal women typically exhibit a higher systemic inflammatory load than their premenopausal counterparts, a phenomenon known as “inflamm-aging” (6). This state not only exponentially increases the risk of metabolic syndrome but also significantly impairs health-related quality of life, necessitating targeted therapeutic strategies (7).

Dysregulation of the “Estrogen-Inflammation Axis” lies at the core of this vicious cycle (8). 17β-estradiol (E2) functions not only as a reproductive hormone but also as a potent immunomodulator. At the molecular level, estrogen inhibits the nuclear translocation of nuclear factor-κB (NF-κB) and blocks macrophage polarization toward the pro-inflammatory M1 phenotype, thereby reducing the production of tissue-derived cytokines (e.g., IL-6, TNF-α) at the source (9). Crucially, the liver—the primary site for synthesizing the systemic inflammatory marker C-reactive protein (CRP)—is highly sensitive to estrogen signaling. Endogenous estrogen acts as a “molecular brake” on the inflammatory cascade by suppressing hepatic responsiveness to IL-6 signaling, thus downregulating CRP synthesis (10). Consequently, the postmenopausal loss of estrogen plunges the body into a unique pathological state characterized by disinhibited hyperactivity of adipose tissue pro-inflammatory secretion and dysregulated hepatic CRP synthesis. Breaking this cycle requires interventions that can effectively mimic or compensate for these lost anti-inflammatory protective mechanisms.

Exercise intervention is recognized as a cornerstone for combating obesity-related inflammation, exerting effects through mechanisms such as inducing myokine secretion, activating the AMP-activated protein kinase (AMPK) pathway, and downregulating Toll-like receptor 4 (TLR4) expression (11, 12). However, despite the established biological plausibility, the optimal exercise prescription for postmenopausal women remains ill-defined. A significant limitation in the current literature is that previous systematic reviews have frequently confounded pre- and postmenopausal populations, obscuring the specific physiological responses driven by the unique hormonal milieu of menopause (13). More critically, the “dose-response” relationships between key exercise characteristics—specifically total volume, intervention duration, and exercise intensity—and inflammatory modulation remain largely unexplored in this specific population. It is currently uncertain whether a minimum threshold of exercise volume is required to trigger anti-inflammatory adaptations in postmenopausal women, or if these benefits accrue linearly with intervention length. Addressing this gap is essential for transitioning from general physical activity guidelines to precision exercise medicine (14).

To address these complexities, this study conducts a systematic review and meta-analysis focusing exclusively on overweight and obese postmenopausal women. Unlike traditional pooled analyses, this study aims to: (1) systematically evaluate the immunometabolic responses to exercise by quantifying changes in systemic (CRP) and tissue-derived (IL-6, TNF-α, adiponectin) inflammatory markers; and (2) explicitly investigate the dose-response relationships between multiple exercise parameters (volume, duration, and intensity) and effect sizes using meta-regression analysis. By clarifying how the “dose” of exercise correlates with the anti-inflammatory “response” in an estrogen-deficient environment, these findings intend to provide evidence-based, precise exercise prescriptions for managing chronic low-grade inflammation in postmenopausal obesity.

## 2 Methods

This systematic review and stratified meta-analysis was conducted in accordance with the Preferred Reporting Items for Systematic Reviews and Meta-Analyses (PRISMA) 2020 statement (15). The study protocol was registered with PROSPERO (registration number: CRD420261288134).

### 2.1 Literature search strategy

A systematic and comprehensive literature search was performed across five major electronic databases: Web of Science, PubMed, the Cochrane Library, MEDLINE, and Embase, covering the period from inception to January 2026. The search strategy was designed to identify randomized controlled trials (RCTs) investigating the dose-response effects of exercise interventions on CLGSI specifically in postmenopausal women with overweight or obesity.

The search algorithm was rigorously structured based on the PICOS framework: (P) Population: postmenopausal women (defined as cessation of menses for >12 months or follicle-stimulating hormone levels consistent with menopause) with overweight or obesity (body mass index [BMI] ≥ 25 kg/m²); (I) Intervention: structured exercise interventions of any modality (e.g., aerobic, resistance, HIIT, or combined training) with a duration of ≥ 8 weeks to ensure sufficient physiological adaptation; (C) Comparator: non-exercise control groups or minimal physical activity groups to isolate the absolute effect of exercise; (O) Outcomes: systemic inflammatory biomarkers (CRP) and tissue-derived cytokines/adipokines (IL-6, TNF-α, adiponectin); and (S) Study Design: randomized controlled trials (RCTs) published in peer-reviewed journals.

Key search terms included combinations of Medical Subject Headings (MeSH) and free-text keywords related to three domains: (1) “Exercise” (e.g., physical activity, aerobic training, resistance training, exercise volume); (2) “Inflammation/Adipokines” (e.g., C-reactive protein, interleukin-6, tumor necrosis factor-alpha, adiponectin); and (3) “Postmenopause/Obesity” (e.g., postmenopausal, obesity, overweight). Boolean operators (AND, OR) were utilized to refine the results. To ensure comprehensive coverage, the reference lists of eligible studies and relevant systematic reviews were manually screened for potential articles not captured by electronic indexing. The detailed search strategies for each database are presented in **Supplementary Table 1**.

### 2.2 Eligibility criteria

Study eligibility was rigorously determined according to the PICOS framework (15), focusing on randomized controlled trials (RCTs) published in English that investigated the effects of structured exercise on inflammatory biomarkers in postmenopausal women. Specifically, inclusion was restricted to studies involving women defined as postmenopausal (cessation of menses for ≥ 12 months or follicle-stimulating hormone levels consistent with menopause, typically > 30 IU/L) in accordance with the STRAW + 10 staging system (16), and classified as overweight or obese (BMI ≥ 25 kg/m² or body fat > 30%) based on World Health Organization guidelines (17). Eligible interventions included aerobic training (AT), resistance training (RT), high-intensity interval training (HIIT), or combined training (CT) with a duration of ≥ 8 weeks to ensure sufficient physiological adaptation (18), compared against non-exercise control groups maintaining habitual lifestyles. Studies were required to report quantitative baseline and post-intervention data (Mean ± SD/SE) for at least one primary outcome (CRP, IL-6, TNF-α, or adiponectin). To isolate the specific effects of exercise and minimize confounding, studies were excluded if participants were current smokers, utilized hormone replacement therapy (HRT) or anti-inflammatory medications (e.g., corticosteroids, NSAIDs), suffered from acute infections or uncontrolled metabolic disorders, or if the intervention involved unequal co-interventions (e.g., dietary restriction or supplements) not matched in the control group (8).

### 2.3 Study selection and data extraction

**Figure 1:**
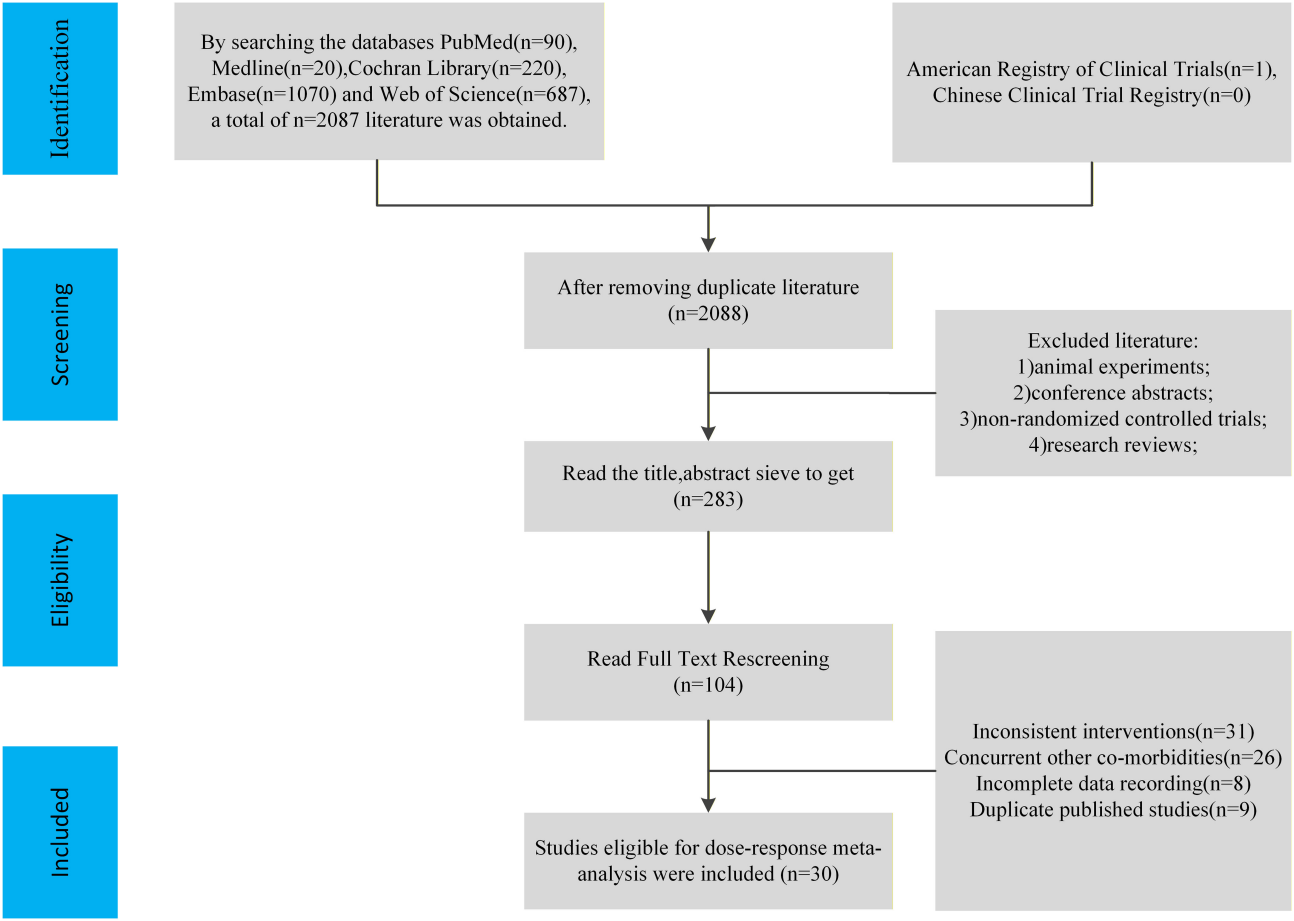
Flow chart of literature screening.

Two investigators independently performed literature screening and data extraction, resolving any discrepancies through discussion or consultation with the wider research team. Following initial title and abstract screening to exclude clearly irrelevant records, potentially eligible studies underwent full-text review to confirm final inclusion (**Figure 1**). Corresponding authors were contacted via email to request supplementary information in cases of missing or incomplete data. Data were extracted using a standardized template, capturing: (1) study characteristics (first author, publication year, country); (2) participant profiles (sample size, age, years since menopause, BMI, body fat percentage); (3) intervention protocols (exercise modality, intensity, session duration, frequency, intervention length, and adherence); and (4) outcome measures (baseline and post-intervention means ± SD/SE for CRP, IL-6, TNF-α, and adiponectin).

To conduct dose-response meta-regression analyses, intervention parameters were systematically standardized into continuous variables. Total exercise volume (MET-minutes/week) was calculated by multiplying the modality-specific metabolic equivalent (MET) value—derived from the Compendium of Physical Activities (19)—by session duration and weekly frequency. Additionally, diverse intensity indicators across trials [e.g., percentage of 1-repetition maximum [%1RM], heart rate reserve [%HRR], or rating of perceived exertion (20)] were converted into a unified metric: estimated percentage of maximum heart rate (%HRmax). These conversions strictly adhered to the established guidelines of the American College of Sports Medicine (ACSM) (21). The detailed conversion logic and the resulting intensity scores for each included trial are presented in **Supplementary Table 2**.

### 2.4 Risk of bias and methodological quality assessment

Two reviewers independently assessed the methodological quality and risk of bias for the 30 included RCTs, strictly in accordance with the Cochrane Handbook (22). The summary of the risk of bias assessment is presented in **Supplementary Figure S1**, and the detailed risk of bias judgments for each included study are shown in **Supplementary Figure S2**.

### 2.5 Data synthesis and statistical analysis

All statistical analyses were conducted using R software (Version 4.3.1) with the ‘meta’ and ‘metafor’ packages (23, 24). Intervention effects were estimated using the Standardized Mean Difference (SMD; Hedges’ g) with 95% confidence intervals (CIs). Effect sizes were calculated based on the changes from baseline to post-intervention in the experimental group versus the control group. When standard deviations (SD) of the change scores were not reported, they were imputed using baseline and post-intervention SDs, assuming a correlation coefficient (r) of 0.5, in accordance with the Cochrane Handbook guidelines (22). A random-effects model (REML method with Hartung-Knapp adjustment) was employed for all pooled analyses to accommodate potential clinical variation, regardless of statistical heterogeneity levels.

Heterogeneity was quantified using the I² statistic and Cochran’s Q test (25), with substantial heterogeneity defined as I² > 50% or P < 0.10. To address the primary research objective regarding the dose-response relationship, random-effects meta-regression analyses were performed. Continuous moderators included total exercise volume (MET-minutes/week), intervention duration (weeks), and exercise intensity (%HRmax) to evaluate whether higher exercise doses or intensities were associated with greater reductions in inflammatory markers.

Sensitivity analyses employed a “leave-one-out” approach to verify the robustness of pooled estimates and ensure that no individual study exerted disproportionate influence on the final results. Publication bias was assessed via visual inspection of funnel plots and Egger’s linear regression test (26). Statistical significance was set at P < 0.05 (two-tailed). The certainty of evidence for the primary outcomes was evaluated using the Grading of Recommendations Assessment, Development and Evaluation (GRADE) framework. Evidence quality was downgraded based on five domains: risk of bias, inconsistency, indirectness, imprecision, and publication bias.

## 3 Results

### 3.1 Characteristics of included studies

This systematic review and meta-analysis included a total of 30 randomized controlled trials (RCTs). The cumulative sample size comprised 2,124 postmenopausal women (experimental group: n = 1,200; control group: n = 924) classified as overweight or obese. The mean age of participants across studies ranged from 54.3 to 88.9 years, reflecting a broad representation of the postmenopausal lifespan. Baseline BMI values spanned 25.1 to approximately 35.0 kg/m², and where body composition was reported, body fat percentages ranged from 33.3% to 56.0%, confirming the consistent adiposity status of the cohorts.

Exercise interventions were categorized into Aerobic Training (AT, n = 16), Resistance Training (RT, n = 9), Combined Training (CT, n = 4), and High-Intensity Interval Training (HIIT, n = 1). Intervention protocols demonstrated considerable variability in dosing, suitable for dose-response analysis: intervention durations ranged from 8 weeks to 12 months, with session frequencies between 2 and 5 sessions per week. Session durations varied significantly, ranging from 30 minutes to 130 minutes. Intervention adherence was generally high, with reported rates ranging from 81% to 100%. Regarding comparator arms, the majority of control groups maintained a habitual sedentary lifestyle or received usual care; notably, five studies utilized a “diet-only” control group to isolate the specific additive effects of exercise beyond caloric restriction.

To construct a comparative model distinguishing “systemic” from “tissue-derived” inflammation, four established biomarkers were selected based on their distinct physiological origins and prognostic value. C-reactive protein (CRP) was utilized as the primary proxy for hepatic-driven systemic inflammation and the acute-phase response (27). In contrast, TNF-α and IL-6 were analyzed to reflect local inflammatory stress originating predominantly from visceral adipose tissue and skeletal muscle (28, 29). Additionally, Adiponectin was included to evaluate the restoration of adipose tissue endocrine function (30), specifically regarding its anti-inflammatory and insulin-sensitizing properties. Detailed characteristics of the included trials are summarized in **Supplementary Table 3**.

### 3.2 Effects of exercise on inflammatory markers and adiponectin

**Figure 2:**
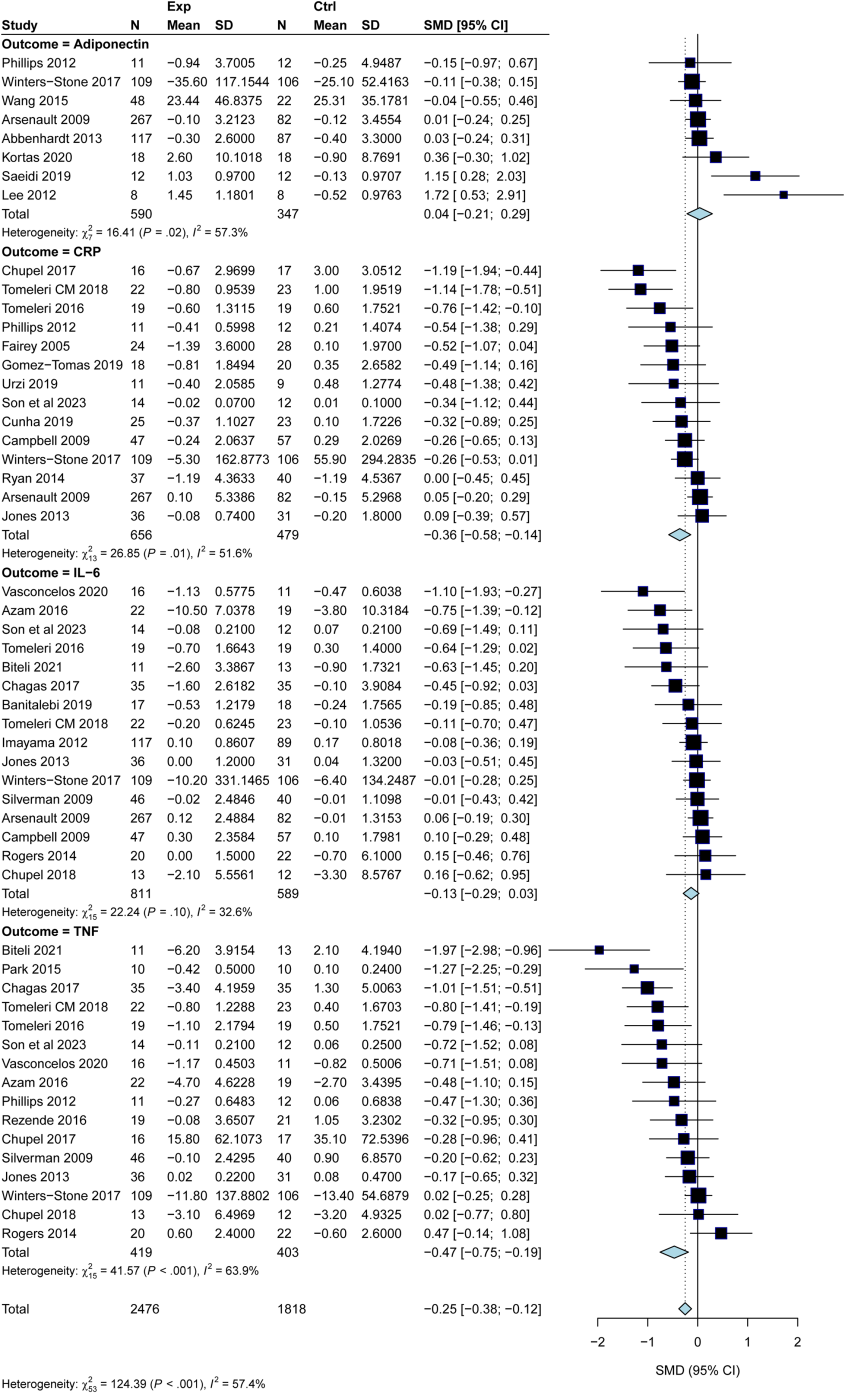
Forest plots summarizing the effects of exercise on inflammatory markers and adiponectin in postmenopausal women. The analysis includes Adiponectin, C-reactive protein (CRP), Interleukin-6 (IL-6), and Tumor necrosis factor-alpha (TNF-α). Data are expressed as standardized mean differences (SMD) with 95% confidence intervals (CI). The red diamonds represent the pooled effect estimates, and the red bars indicate the prediction intervals. I² indicates the level of statistical heterogeneity.

**Figure 2** summarizes the aggregated meta-analysis results for the four circulating biomarkers. Overall, exercise interventions demonstrated differential effects on the inflammatory profile of postmenopausal women. Significant reductions were observed for TNF-α and CRP. TNF-α showed the largest reduction (SMD = -0.47; 95% CI: -0.75 to -0.19; p < 0.001), followed by a moderate decrease in CRP levels (SMD = -0.36; 95% CI: -0.58 to -0.14; p = 0.001). In contrast, changes in IL-6 (SMD = -0.13; 95% CI: -0.29 to 0.03; p = 0.10) and Adiponectin (SMD = 0.04; 95% CI: -0.21 to 0.29; p = 0.76) were not statistically significant, although IL-6 trended downwards. Moderate-to-high heterogeneity was detected across the analyses (I² range: 32.6% to 63.9%). Notably, the prediction intervals for all markers, including CRP and TNF-α, crossed the line of null effect. This indicates that while exercise is generally effective for reducing systemic inflammation, the magnitude of clinical benefit may vary depending on individual patient characteristics or specific intervention protocols.

### 3.3 Sensitivity analysis

To assess the stability of the pooled effect sizes, we conducted a “leave-one-out” sensitivity analysis. For CRP (**Supplementary Figure S3A**), the overall significant reduction demonstrated remarkable robustness. Excluding any individual trial, including the heavily weighted trials by Campbell et al. (31) or Winters-Stone et al. (32), did not alter the statistical significance of the pooled effect (SMD range: -0.41 to -0.28), indicating that the primary findings for CRP are stable and not driven by a single study. For IL-6 (**Supplementary Figure S3B**), the effect sizes remained directionally consistent but statistically non-significant across all iterations (SMD range: -0.17 to -0.09). In contrast, TNF-α (**Supplementary Figure S3C**) demonstrated robust stability; the significant reduction was maintained regardless of any single study exclusion (SMD range: -0.52 to -0.40). Finally, the analysis for adiponectin (**Supplementary Figure S3D**) showed consistent non-significance (SMD range: 0.01 to 0.25), confirming that exercise does not independently alter this biomarker within the current dataset.Furthermore, varying the imputed correlation coefficient (r = 0.3 and r = 0.7) for missing SDs did not alter the direction or significance of the pooled estimates.

### 3.4 Dose-response analysis

We performed meta-regression analyses to determine whether exercise characteristics—specifically total exercise volume (MET-min/week), intervention duration (weeks), and exercise intensity (%HRmax)—predict the magnitude of anti-inflammatory effects. The regression results are detailed in **Supplementary Figures S4–S6**. For total exercise volume (**Supplementary Figure S4**), we observed no statistically significant linear associations across the biomarkers. Although CRP (p = 0.089) and TNF-α (p = 0.092) showed borderline trends, neither reached statistical significance (p < 0.05). Similarly, intervention duration (**Supplementary Figure S5**) was not a significant predictor of effect size for CRP (p = 0.544), IL-6 (p = 0.990), TNF-α (p = 0.610), or adiponectin (p = 0.677). Regarding exercise intensity (**Supplementary Figure S6**), a borderline trend was noted for TNF-α (p = 0.063), suggesting a potential association between higher intensity and larger effect sizes, though this fell short of statistical significance. No linear relationships were found for CRP (p = 0.261), IL-6 (p = 0.537), or adiponectin (p = 0.396). Overall, within the parameters of the included studies, our analysis did not establish a definitive linear dose-response relationship for exercise volume, duration, or intensity across these inflammatory markers.

### 3.5 Publication bias

**Figure 3:**
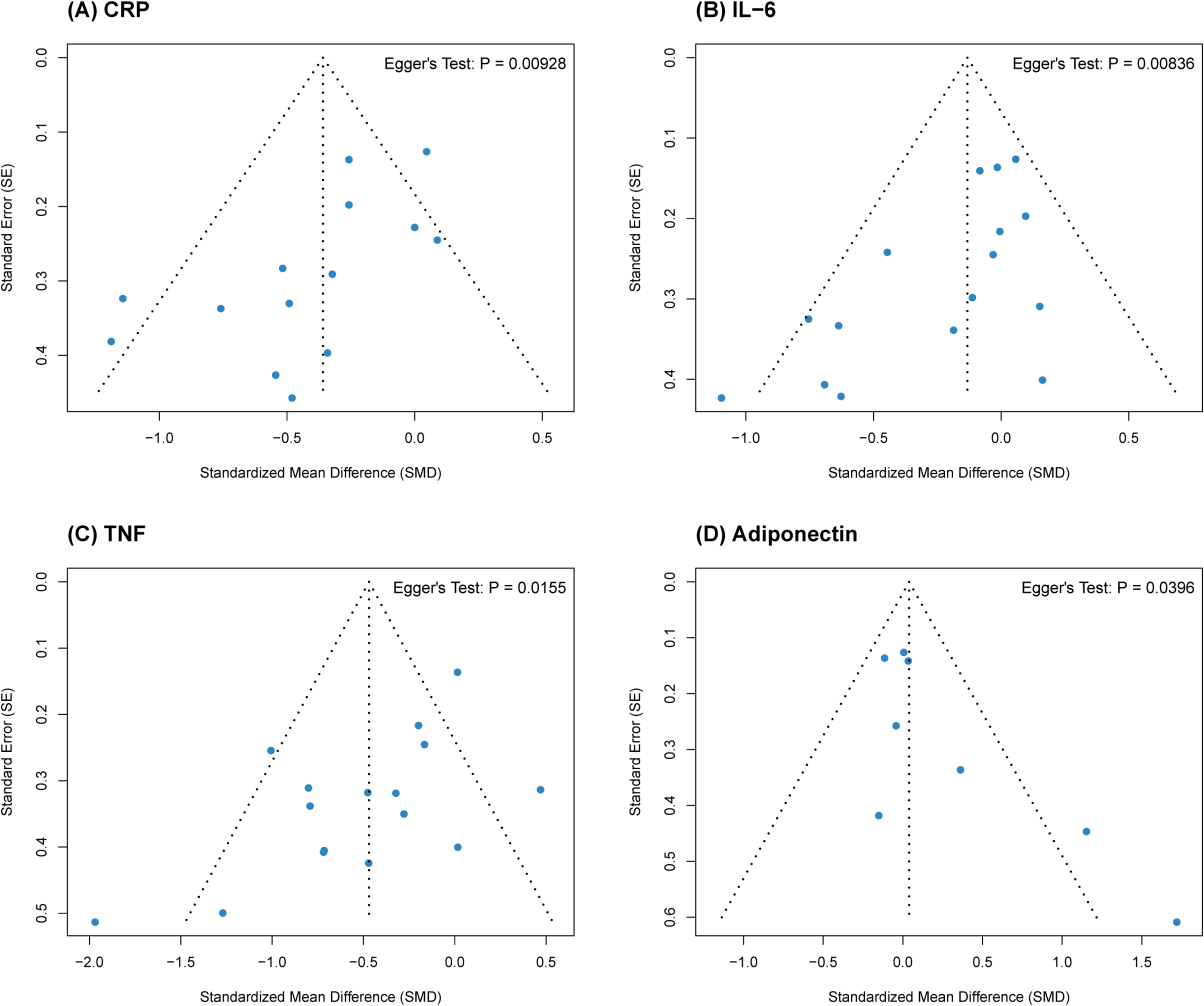
Funnel plots for publication bias assessment. The analysis includes **(A)** C-reactive protein (CRP), **(B)** Interleukin-6 (IL-6), **(C)** Tumor necrosis factor-alpha (TNF-α), and **(D)** Adiponectin. The blue dots represent individual studies, the dotted lines indicate the 95% pseudo-confidence intervals, and Egger's test p-values are provided to indicate potential publication bias.

Potential publication bias was assessed through visual inspection of funnel plots (**Figure 3**) and quantified using Egger’s linear regression test (26). The analysis indicated statistically significant asymmetry across all four biomarkers (Adiponectin p = 0.040; CRP p = 0.009; IL-6 p = 0.008; TNF-α p = 0.016).

While such asymmetry is canonically interpreted as evidence of publication bias, the scatter observed in these plots, when interpreted alongside the heterogeneity noted earlier, suggests a likely contribution from “small-study effects” (33). This indicates that smaller trials in this analysis tended to report larger effect sizes compared to larger studies. Such a pattern may stem from methodological differences—such as higher intervention intensity or stricter supervision in smaller cohorts—rather than solely from the suppression of non-significant findings (34). Consequently, while the aggregate results support the anti-inflammatory benefits of exercise, the influence of these smaller studies on the overall effect magnitude should be acknowledged.

### 3.6 Certainty of evidence

The overall certainty of evidence for the outcomes was evaluated using the GRADE framework (35), with detailed assessments provided in **Supplementary Table 4**. The certainty of evidence for the significant reductions in CRP and TNF-α was rated as “Low”. Although the synthesized data were derived exclusively from RCTs, downgrades were necessary due to serious inconsistency (moderate-to-high statistical heterogeneity) and publication bias (small-study effects). The certainty of evidence for the non-significant changes in IL-6 and adiponectin was rated as “Very Low,” following an additional downgrade for imprecision, as their wide confidence intervals crossed the line of null effect.

## 4 Discussion

This meta-analysis is the first to systematically evaluate the dose-response relationships between diverse exercise parameters (volume, duration, intensity) and inflammatory modulation exclusively in overweight and obese postmenopausal women. By synthesizing data from 30 randomized controlled trials, we demonstrated that exercise interventions effectively mitigate both systemic (CRP) and tissue-derived (TNF-α) inflammation in this estrogen-deficient population. Notably, our meta-regression challenges the traditional “more is better” paradigm, revealing the absence of definitive linear dose-response relationships for total exercise volume and duration. Instead, a borderline trend (p = 0.063) for TNF-α in response to higher exercise intensity emerged, highlighting intensity as a potentially critical regulator of adipose-derived inflammatory pathways. These findings suggest that precision exercise prescriptions for postmenopausal obesity should prioritize specific intensity thresholds rather than merely amassing total physical activity volume.

### 4.1 Efficacy of exercise in an estrogen-deficient environment

The transition through menopause creates a “second hit” to metabolic health, characterized by estrogen withdrawal, visceral adiposity, and a hyperactive inflammatory cascade (4, 5). Our findings confirm that structured exercise significantly attenuates this “inflamm-aging” process, as evidenced by the robust reductions in CRP (SMD = -0.36) and TNF-α (SMD = -0.47). Mechanistically, in the absence of the 17β-estradiol (E2) “molecular brake,” the liver becomes hyper-responsive to circulating cytokines, dysregulating CRP synthesis (10). Exercise effectively mimics the lost anti-inflammatory protection of estrogen by activating the hepatic AMPK-SIRT1 pathway, which suppresses lipotoxicity-induced Kupffer cell activation and subsequent NF-κB signaling (36). Concurrently, the significant reduction in TNF-α indicates that skeletal muscle contraction successfully antagonizes local adipose tissue inflammation. This muscle-adipose cross-talk, primarily mediated by exercise-induced myokines, effectively mitigates the hypertrophic and pro-inflammatory secretory profile of visceral fat, thereby breaking the “menopause-obesity-inflammation” vicious cycle (11, 12).

### 4.2 The crucial role of exercise intensity over volume

A central objective of this study was to adjudicate the optimal “dose” of exercise. Surprisingly, neither total exercise volume (MET-min/week) nor intervention duration demonstrated significant linear associations with the reduction of any inflammatory marker. This indicates a “threshold effect” rather than a continuous dose-response accumulation; once a minimum physiological stimulus is achieved (e.g., standard interventions lasting ≥ 8 weeks), extending the duration or amassing excess volume does not linearly yield greater anti-inflammatory dividends.

Conversely, the meta-regression identified a notable borderline trend (p = 0.063) indicating that higher exercise intensity is associated with greater reductions in TNF-α. This aligns with the “intensity threshold” hypothesis. Mechanistically, vigorous exercise induces exponentially greater vascular shear stress, heavily downregulating pro-inflammatory endothelial signaling. Furthermore, higher-intensity protocols elicit a superior catecholamine response, driving enhanced lipolysis and oxidation specifically within visceral adipose tissue, which is the primary source of TNF-α. For clinical practice, this implies that for postmenopausal women capable of tolerating the physical demand, incrementally increasing exercise intensity may offer a more potent and time-efficient therapeutic strategy than simply prolonging low-intensity sessions (21).

### 4.3 Stability of findings and methodological considerations

The robustness of our primary findings is unequivocally confirmed by the rigorous leave-one-out sensitivity analyses. For both systemic (CRP) and tissue-derived (TNF-α) inflammatory markers, the significant reductions remained statistically stable across all iterations. Specifically, the sequential exclusion of any single trial—even heavily weighted studies—failed to shift the confidence intervals across the line of null effect (CRP SMD range: -0.41 to -0.28; TNF-α SMD range: -0.52 to -0.40). This exceptional resilience demonstrates that the anti-inflammatory efficacy of exercise is a generalized physiological adaptation in postmenopausal women, rather than a statistical artifact driven by isolated large-scale or extreme-outcome studies. Similarly, the consistent non-significance of IL-6 and adiponectin across all permutations reinforces the reliability of these specific null findings.

However, we acknowledge the presence of publication bias, as evidenced by asymmetrical funnel plots and significant Egger’s tests across all biomarkers. When interpreted alongside the detected moderate-to-high heterogeneity, this asymmetry is highly indicative of “small-study effects” (33). Smaller trials in exercise science frequently employ higher relative intervention intensities or maintain stricter supervised compliance, thereby reporting larger effect sizes compared to massive, loosely supervised multi-center trials (34). Thus, while these smaller studies may slightly inflate the aggregate effect magnitude, the unwavering stability of the sensitivity analysis confirms the fundamental clinical efficacy of exercise.

### 4.4 Strengths and limitations

The principal strength of this review is its strict adherence to a specific physiological demographic (overweight and obese postmenopausal women) and the rigorous quantification of diverse intensity metrics into a standardized continuous variable (%HRmax). Nevertheless, several limitations warrant consideration. First, IL-6 and adiponectin did not exhibit significant changes. The modulation of adiponectin, in particular, may require more substantial, concurrent weight loss rather than exercise alone. Second, our dose-response meta-regression analyses were univariate. Because higher-intensity interventions are occasionally prescribed with shorter durations or specific training modalities, we cannot completely rule out the potential synergistic or confounding effects among these variables. Third, while we standardized exercise intensity, the distinct physiological adaptations between pure resistance training and aerobic training might inherently differ, contributing to the observed heterogeneity. Fourth, although 30 RCTs were included overall, the number of studies reporting specific biomarkers (e.g., only 8 trials for adiponectin) limited the statistical power of the meta-regression analyses for these outcomes, increasing the risk of Type II errors. Future individual participant data (IPD) meta-analyses and multivariate models are required to further untangle the independent and synergistic effects of exercise intensity and modality.

## 5 Conclusion

In conclusion, structured exercise functions as a potent, precision therapeutic modality for postmenopausal women, essential for dismantling the “menopause-obesity-inflammation” axis. Our analysis confirms that exercise effectively and reliably mitigates both systemic (CRP) and tissue-derived (TNF-α) inflammation in an estrogen-deficient environment. Crucially, sensitivity analyses prove that these immunometabolic benefits are exceptionally stable and generalizable, independent of any single trial’s influence. Furthermore, our dose-response analyses challenge the traditional reliance on total exercise volume or duration as primary targets. Instead, the data point toward an “intensity threshold,” suggesting that higher exercise intensities more effectively drive the reduction of adipose-derived TNF-α. Future clinical guidelines should emphasize the optimization of exercise intensity to maximize immunometabolic rescue in postmenopausal obesity.

## Data Availability

The original contributions presented in the study are included in the article/**Supplementary Material**. Further inquiries can be directed to the corresponding author.
