# Supplementary material for: Effects of exercise intensity and volume on systemic inflammation in overweight and obese postmenopausal women: a dose-response meta-analysis
Source: Front Immunol. 2026 Mar 19;17:1801309. doi: 10.3389/fimmu.2026.1801309 (PMC13043358; doi:10.3389/fimmu.2026.1801309)
Supplement: Supplementary file 1 [file DataSheet1.docx]

**Supplemental material**

| **Content** | **Pages** |
| --- | --- |
| **Table S1.** Search strategy | 2-3 |
| **Table S2.** Detailed Conversion Logic of Exercise Intensity for Each Included Study | 4-5 |
| **Table S3.** Characteristics of included studies | 6-7 |
| **Figure S1.** Summary of the risk of bias assessment | 8 |
| **Figure S2.** Risk of bias assessment results | 9 |
| **Figure S3.** Leave-one-out sensitivity analysis for the inflammatory markers and adiponectin. | 10 |
| **Figure S4.** Dose-response meta-regression analysis of total exercise volume. | 10 |
| **Figure S5.** Dose-response meta-regression analysis of intervention duration. | 11 |
| **Figure S6.** Dose-response meta-regression analysis of exercise intensity. | 11 |
| **Table S4.** GRADE certainty of evidence for the primary outcomes | 12 |
| **Table S5.** PRISMA meta-analysis checklist | 13-14 |

**Table S1.** Search strategy

| Database | Search strategy |
| --- | --- |
| Pubmed (90) | ("Exercise"[Mesh] OR "Physical Exertion"[Mesh] OR "Sports"[Mesh] OR "exercise"[Title/Abstract] OR "exercises"[Title/Abstract] OR "physical activity"[Title/Abstract] OR "sport"[Title/Abstract] OR "sports"[Title/Abstract] OR "training"[Title/Abstract] OR "aerobic"[Title/Abstract] OR "resistance"[Title/Abstract] OR "strength"[Title/Abstract] OR "combined training"[Title/Abstract] OR "concurrent training"[Title/Abstract] OR "interval training"[Title/Abstract] OR "HIIT"[Title/Abstract] OR "sprint"[Title/Abstract]) AND ("Obesity"[Mesh] OR "Overweight"[Mesh] OR "Body Mass Index"[Mesh] OR "obesity"[Title/Abstract] OR "obese"[Title/Abstract] OR "overweight"[Title/Abstract] OR "body mass index"[Title/Abstract] OR "BMI"[Title/Abstract] OR "fat mass"[Title/Abstract] OR "adiposity"[Title/Abstract]) AND ("Postmenopause"[Mesh] OR "postmenopause"[Title/Abstract] OR "postmenopausal"[Title/Abstract] OR "post-menopause"[Title/Abstract] OR "post-menopausal"[Title/Abstract]) AND ("Inflammation"[Mesh] OR "Cytokines"[Mesh] OR "C-Reactive Protein"[Mesh] OR "Interleukin-6"[Mesh] OR "Tumor Necrosis Factor-alpha"[Mesh] OR "Adiponectin"[Mesh] OR "inflammation"[Title/Abstract] OR "inflammatory"[Title/Abstract] OR "cytokine*"[Title/Abstract] OR "C-reactive protein"[Title/Abstract] OR "CRP"[Title/Abstract] OR "Interleukin-6"[Title/Abstract] OR "IL-6"[Title/Abstract] OR "Tumor Necrosis Factor-alpha"[Title/Abstract] OR "TNF-alpha"[Title/Abstract] OR "TNF-α"[Title/Abstract] OR "adiponectin"[Title/Abstract] OR "adipokine*"[Title/Abstract]) AND ("Randomized Controlled Trial"[Publication Type] OR "Randomized Controlled Trials as Topic"[Mesh] OR "randomized controlled trial"[Title/Abstract] OR "randomised controlled trial"[Title/Abstract] OR "RCT"[Title/Abstract] OR "randomized"[Title/Abstract] OR "randomised"[Title/Abstract] OR "placebo"[Title/Abstract]) |
| Cochrane (220) | #1 MeSH descriptor: [Exercise] explode all trees 42,079  #2 (sport*):ti,ab,kw or (train*):ti,ab,kw or (physical activity):ti,ab,kw or (aerobic exercise):ti,ab,kw or (aerobic training):ti,ab,kw or (moderate intensity continuous training):ti,ab,kw or (resistance training):ti,ab,kw or (resistance exercise):ti,ab,kw or (strength training):ti,ab,kw or (strength exercise):ti,ab,kw or (combined training):ti,ab,kw or (combined exercise):ti,ab,kw or (concurrent training):ti,ab,kw or (high intensity interval training):ti,ab,kw or (sprint interval training):ti,ab,kw or (low intensity exercise):ti,ab,kw or (low intensity training):ti,ab,kw or (moderate intensity exercise):ti,ab,kw or (moderate intensity training):ti,ab,kw or (high intensity exercise):ti,ab,kw or (high intensity training):ti,ab,kw or (sprint training):ti,ab,kw or (sprint exercise):ti,ab,kw 260,825  #3 MeSH descriptor: [Obesity] explode all trees 22,903  #4 MeSH descriptor: [Overweight] explode all trees 26,703  #5 MeSH descriptor: [Women] explode all trees 1561  #6 MeSH descriptor: [Female] explode all trees 634,167  #7 MeSH descriptor: [Inflammation] explode all trees 17,033  #8 (inflammatory reaction):ti,ab,kw or (inflammatory factors):ti,ab,kw or (inflammatory markers):ti,ab,kw or (inflammatory response):ti,ab,kw or (inflammatory cytokine):ti,ab,kw or (inflammatory cytokines):ti,ab,kw 51,366  #9 #1 OR #2 269,692  #10 #3 OR #4 26,703  #11 #5 OR #6 634,184  #12 #7 OR #8 63,094  #13 #9 AND #10 AND #11 AND #12 220 |
| Embase (1,070) | ('exercise'/exp OR 'sport'/exp OR 'physical activity'/exp OR 'kinesiotherapy'/exp OR 'resistance training'/exp OR 'aerobic exercise'/exp OR 'endurance training'/exp OR 'high intensity interval training'/exp OR exercise*:ti,ab,kw OR 'physical activity':ti,ab,kw OR sport*:ti,ab,kw OR training:ti,ab,kw OR aerobic:ti,ab,kw OR resistance:ti,ab,kw OR strength:ti,ab,kw OR 'combined training':ti,ab,kw OR 'concurrent training':ti,ab,kw OR 'interval training':ti,ab,kw OR hiit:ti,ab,kw OR sprint:ti,ab,kw) AND ('obesity'/exp OR 'body mass'/exp OR 'adipose tissue'/exp OR obesity:ti,ab,kw OR obese:ti,ab,kw OR overweight:ti,ab,kw OR 'body mass index':ti,ab,kw OR bmi:ti,ab,kw OR 'fat mass':ti,ab,kw OR adiposity:ti,ab,kw) AND ('postmenopause'/exp OR postmenopaus*:ti,ab,kw OR 'post menopause':ti,ab,kw OR 'post-menopause':ti,ab,kw OR 'post menopaus*':ti,ab,kw) AND ('inflammation'/exp OR 'cytokine'/exp OR 'c reactive protein'/exp OR 'interleukin 6'/exp OR 'tumor necrosis factor alpha'/exp OR 'adiponectin'/exp OR inflammation:ti,ab,kw OR inflammatory:ti,ab,kw OR cytokine*:ti,ab,kw OR 'c-reactive protein':ti,ab,kw OR crp:ti,ab,kw OR 'interleukin-6':ti,ab,kw OR 'il-6':ti,ab,kw OR 'tumor necrosis factor-alpha':ti,ab,kw OR 'tnf-alpha':ti,ab,kw OR 'tnf-a':ti,ab,kw OR adiponectin:ti,ab,kw OR adipokine*:ti,ab,kw) AND ('randomized controlled trial'/exp OR 'clinical trial'/exp OR 'controlled clinical trial'/exp OR 'randomized controlled trial':ti,ab,kw OR 'controlled clinical trial':ti,ab,kw OR randomized:ti,ab,kw OR placebo:ti,ab,kw OR 'drug therapy':ti,ab,kw OR randomly:ti,ab,kw OR trial:ti,ab,kw OR groups:ti,ab,kw) NOT ('conference abstract'/it OR 'conference paper'/it OR 'editorial'/it OR 'letter'/it OR 'note'/it) 1,070 |
| Web of Science  (687) | TS=(exercise* OR "physical activity" OR sport* OR training OR aerobic OR resistance OR strength OR "combined training" OR "concurrent training" OR "interval training" OR HIIT OR sprint*) AND TS=(obesity OR obese OR overweight OR "body mass index" OR BMI OR "fat mass" OR adiposity) AND TS=(postmenopaus* OR "post menopause" OR "post-menopause" OR post-menopaus*) AND TS=(inflammation OR inflammatory OR cytokine* OR "C-reactive protein" OR CRP OR "Interleukin-6" OR "IL-6" OR "Tumor Necrosis Factor-alpha" OR "TNF-alpha" OR "TNF-a" OR adiponectin OR adipokine*) AND TS=("randomized controlled trial" OR "controlled clinical trial" OR randomized OR placebo OR "drug therapy" OR randomly OR trial OR groups) |
| MEDLINE (20) | ((MeSH descriptor: [Exercise] explode all trees) OR (MeSH descriptor: [Sports] explode all trees) OR (MeSH descriptor: [Physical Exertion] explode all trees) OR ((exercise* OR "physical activity" OR sport* OR training OR aerobic OR resistance OR strength OR "combined training" OR "concurrent training" OR "interval training" OR HIIT OR sprint):ti,ab,kw)) AND ((MeSH descriptor: [Obesity] explode all trees) OR (MeSH descriptor: [Overweight] explode all trees) OR (MeSH descriptor: [Body Mass Index] explode all trees) OR ((obesity OR obese OR overweight OR "body mass index" OR BMI OR "fat mass" OR adiposity):ti,ab,kw)) AND ((MeSH descriptor: [Postmenopause] explode all trees) OR ((postmenopaus* OR "post menopause" OR "post-menopause" OR post-menopaus*):ti,ab,kw)) AND ((MeSH descriptor: [Inflammation] explode all trees) OR (MeSH descriptor: [Cytokines] explode all trees) OR (MeSH descriptor: [C-Reactive Protein] explode all trees) OR (MeSH descriptor: [Interleukin-6] explode all trees) OR (MeSH descriptor: [Tumor Necrosis Factor-alpha] explode all trees) OR (MeSH descriptor: [Adiponectin] explode all trees) OR ((inflammation OR inflammatory OR cytokine* OR "C-reactive protein" OR CRP OR "Interleukin-6" OR "IL-6" OR "Tumor Necrosis Factor-alpha" OR "TNF-alpha" OR "TNF-a" OR adiponectin OR adipokine*):ti,ab,kw)) AND ((MeSH descriptor: [Randomized Controlled Trial] explode all trees) OR (("randomized controlled trial" OR "controlled clinical trial" OR randomized OR placebo OR "drug therapy" OR randomly OR trial OR groups):ti,ab,kw)) |

**Table S2.** Detailed Conversion Logic of Exercise Intensity for Each Included Study

| Study (Year) | Original Exercise Modality & Reported Intensity | Standardized %HRmax | Conversion Logic / Justification (ACSM Guidelines) |
| --- | --- | --- | --- |
| Abbenhardt (2013) | Aerobic; 60–85% HRmax | 72.5 | Direct midpoint imputation of the 60–85% range. |
| Arsenault (2009) | Aerobic; 50% & 75% VO2peak | 75 | Mapped to ACSM Moderate-to-Vigorous equivalent; assigned 75% to reflect the higher target intensity group. |
| Azam (2016) | HIIT; High Intensity Interval | 90 | Qualitative conversion; HIIT is canonically defined as near-maximal to maximal (≥90% HRmax). |
| Banitalebi (2019) | Combined; Moderate | 65 | Qualitative conversion; ACSM Moderate category spans 64–76% HRmax; assigned lower bound/midpoint. |
| Biteli (2021) | Combined; Circuit Training, RPE 15–17 | 85 | ACSM RPE mapping; RPE 14–17 corresponds to Vigorous (77–95% HRmax); assigned midpoint 85%. |
| Campbell (2009) | Aerobic; 60–75% HRR | 80 | ACSM HRR mapping; 60-89% HRR corresponds to Vigorous (77–95% HRmax); assigned representative 80%. |
| Chagas (2017) | Resistance; 8–12 RM (to failure) | 80 | ACSM Vigorous equivalence; training to failure at 8-12 RM elicits high relative cardiovascular stress. |
| Chupel (2017) | Combined; Moderate-to-High | 75 | Qualitative mapping; assigned 75% as the boundary between Moderate (≤76%) and Vigorous (≥77%). |
| Chupel (2018) | Combined; Moderate-to-High | 75 | Qualitative mapping; assigned boundary value of 75%. |
| Cunha (2019) | Resistance; 60–80% 1RM | 70 | ACSM Resistance-to-Aerobic equivalence; mapped to 70% HRmax. |
| Fairey (2005) | Aerobic; 70–75% HRpeak | 72.5 | Direct midpoint imputation of the 70–75% range. |
| Gomez-Tomas (2019) | Aerobic; Brisk Walking, RPE 12–14 | 65 | ACSM RPE mapping; RPE 12–13 is Moderate (64–76% HRmax); assigned 65%. |
| Imayama (2012) | Aerobic; 50% & 75% VO2max | 75 | ACSM VO2max mapping; 75% VO2max corresponds to ~80% HRmax; averaged/assigned 75% for overall cohort. |
| Jones (2013) | Aerobic; 65–75% HRmax | 70 | Direct midpoint imputation of the 65–75% range. |
| Kortas (2020) | Aerobic; Nordic Walking, 60–70% HRmax | 65 | Direct midpoint imputation of the 60–70% range. |
| Lee (2012) | Aerobic; Walking, 50–60% HRR | 70 | ACSM HRR mapping; 40–59% HRR is Moderate (64–76% HRmax); assigned midpoint 70%. |
| Park (2015) | Aerobic; Treadmill walking, 60% HRR | 75 | ACSM HRR mapping; 60% HRR borders the Vigorous category (≥77% HRmax); conservatively assigned 75%. |
| Phillips (2012) | Aerobic; 65–85% HRR | 85 | ACSM HRR mapping; 65–85% HRR is strictly Vigorous; assigned midpoint equivalence 85%. |
| Rezende (2016) | Aerobic; Walking, 50–70% HRR | 75 | ACSM HRR mapping; spans Moderate to Vigorous; assigned 75% HRmax equivalence. |
| Rogers (2014) | Aerobic; Walking, RPE 11–13 | 60 | ACSM RPE mapping; RPE 11 is Light, 12-13 is Moderate; assigned borderline value 60%. |
| Ryan (2014) | Aerobic; Walking, 50–60% VO2peak | 70 | ACSM VO2peak mapping; 40–59% VO2R is Moderate (64–76% HRmax); assigned 70%. |
| Saeidi (2019) | Resistance; Progressive, Mod-High | 70 | Qualitative mapping; assigned Moderate-to-Vigorous transition value (70%). |
| Silverman (2009) | Aerobic; Walking/Jogging, Mod-Vigorous | 70 | Qualitative mapping; assigned midpoint spanning Moderate and Vigorous descriptions. |
| Son et al. (2023) | Combined; Pilates and Aerobic, Moderate | 60 | Qualitative mapping; conservative assignment at the lower boundary of Moderate. |
| Tomeleri (2016) | Resistance; 10–15 RM | 80 | ACSM Vigorous equivalence; high-effort 10-15 RM corresponds to high relative intensity. |
| Tomeleri CM (2018) | Resistance; 10–15 RM | 80 | ACSM Vigorous equivalence; high-effort 10-15 RM corresponds to high relative intensity. |
| Urzi (2019) | Resistance; 60–80% 1RM | 70 | ACSM Resistance-to-Aerobic equivalence; 60-80% 1RM aligns with Moderate-to-Vigorous HRmax. |
| Vasconcelos (2020) | Aerobic; Walking, 50–70% HRmax | 60 | Direct midpoint imputation of the 50–70% range. |
| Wang (2015) | Aerobic; 45–50% & 70–75% VO2max | 73 | Weighted average of the two reported VO2max intensity tiers mapped to HRmax. |
| Winters-Stone (2017) | Resistance; Weighted vest, 60–80% 1RM | 70 | ACSM Resistance-to-Aerobic equivalence; mapped to 70% HRmax. |

Note: The standard conversions strictly adhered to the ACSM Position Stand (Garber et al., 2011). Imputations were made using the midpoint of reported ranges or by mapping equivalent physiological categories (e.g., %1RM or RPE to %HRmax).

**Table S3.** Characteristics of included studies

| **Study** | **Country** | **Participant Characteristics (Exp/Ctrl)** | | | | | **Intervention Protocol (Exp Group)** | | | **Adherence (%)** | **Ctrl Activity** | **Outcomes** |
| --- | --- | --- | --- | --- | --- | --- | --- | --- | --- | --- | --- | --- |
|  |  | **N** | **Age(years)** | **BMI (kg/m²)** | **Fat Mass (%)** | **Weight Status** | **Type** | **Intensity** | **Duration/Freq** |  |  |  |
| Abbenhardt et al., 2013 | USA | 117 / 87 | 58.1 ± 5.0 / 57.4 ± 4.4 | > 25 / > 25 | – | OB/OW | AT | 70%–85% HRmax | 45 min/d, 5 x/wk, 12 mo | 86% | Habitual Lifestyle | Adiponectin |
| Arsenault et al., 2009 | USA | 267 / 82 | 57.3 ± 6.6 / 57.2 ± 6.1 | 32.0 ± 5.7 /  31.9 ± 3.8 | – | OB/OW | AT | 50% VO_2_peak | 3–4 x/wk, 6 mo | 92.20% | Non-exercise / Sedentary | Adiponectin, CRP, IL-6 |
| Azam et al., 2016 | Iran | 22 / 19 | 58.8 ± 6.4 / 55.3 ± 5.2 | 28.2 ± 3.4 /  29.9 ± 2.3 | 42.5 ± 2.4 /  40.1 ± 4.3 | OB | AT | 70%– 80% HRmax | 50 min/d, 3 x/wk, 6 mo | NR (>80% implied) | Sedentary / Usual Lifestyle | IL-6, TNF-α |
| Banitalebi et al., 2019 | Iran | 17 / 18 | 55.36 ± 5.94 /  55.71 ± 6.40 | 29.27 ± 3.00 / 30.12 ± 3.52 | 41.14 ± 4.34 / 42.64 ± 4.95 | OB/OW | HIIT | 4 × 30 s intervals (maximum intensity) | 50 min/d, 3 x/wk, 10 wks | 100% (Reported) | No training / Sedentary | IL-6 |
| Biteli et al., 2021 | Brazil | 11 / 13 | 58.5 ± 6.5 / 61.2 ± 7.7 | – | 56 ± 2 /  55.7 ± 4 | OB | AT | 50%–60% HRR | 75 min/d, 3 x/wk, 20 wks | > 85% | Sedentary (No exercise) | IL-6, TNF-α |
| Campbell et al., 2009 | USA | 53 / 62 | 60.5 ± 7.0 / 60.9 ± 6.8 | 30.2 ± 4.0 /  30.4 ± 3.8 | – | OB/OW | AT | 60%–75% HRmax | 45 min/d, 3 x/wk, 12 wks | 86% | Habitual Lifestyle | CRP, IL-6 |
| Chagas et al., 2017 | Brazil | 44 / 38 | 61.3 ± 6.4 / 59.8 ± 7.1 | – | – | OW | AT | 50%–60% VO_2_peak | 75 min/d, 3 x/wk, 20 wks | 85.50% | No exercise / Habitual routine | IL-6, TNF-α |
| Chupel et al., 2017 | Portugal | 16 / 17 | 83.5 ± 5.13 / 82.12 ± 6.41 | 29.27 ± 7.10 / 29.67 ± 5.98 | – | OB/OW | RT | 6–8 OMNI–GSE | 45 min, 2–3 x/wk, 28 wks | 82% | Sedentary | CRP, TNF-α |
| Chupel et al., 2018 | Portugal | 13 / 12 | 83.5 ± 7.3 / 82 ± 7.5 | 27.2 ± 3.8 /  30.3 ± 3.5 | – | OB/OW | CT | 5–7 OMNI–GSE | 60 min/d, 2 x/wk, 14 wks | 85% | Sedentary + Placebo | IL-6, TNF-α |
| Cunha et al., 2019 | Portugal | 25 / 23 | 71.40 ± 5.71 / 69.04 ± 4.45 | 26.39 ± 4.55 / 28.26 ± 5.01 | – | OB/OW | RT | 10–15 RM, 10–15 rep/sets | 3 x/wk, 12 wks | > 85% | No exercise | CRP |
| Fairey et al., 2005 | Canada | 24 / 28 | 59 ± 5 /  58 ± 6 | 29.4 ± 7.4 /  29.1 ± 6.1 | – | OB/OW | AT | 70%–75% VO_2_peak | 30 min/d, 3 x/wk, 15 wks | 83% | Usual Care | CRP |
| Gomez-Tomas et al., 2019 | Iran | 18 / 20 | 70.89 ± 4.42 / 70.45 ± 5.44 | 28.72 ± 4.48 / 30.16 ± 5.57 | – | OB/OW | RT | mo 1–4: 3–4 OMNI-RES; mo 5–8: 5–6 OMNI-RES; mo 9–12: 6–7 OMNI-RES | 50 min/d, 3 x/wk, 12 mo | 92% | Daily activities (No exercise) | CRP |
| Imayama et al., 2012 | USA | 117 / 89 | 58.1 ± 5.0 / 57.4 ± 4.4 | 30.7 ± 3.7 /  30.7 ± 3.9 | – | OB/OW | AT | 70%–85% HRmax | 225 min/wk, 5 x/wk, 12 wks | 86% (Ex), 93% (Diet) | Habitual Lifestyle | IL-6 |
| Jones et al., 2013 | USA | 36 / 31 | 56.4 ± 9.6 / 55.4 ± 7.6 | 29.4 ± 7.3 /  30.6 ± 6.0 | – | OB/OW | AT | 60%–80% HRmax | 120 min/wk, 6 mo | 81% | Usual Care | CRP, IL-6, TNF-α |
| Kortas et al., 2020 | Poland | 18 / 18 | 66.78 ± 4.76 / 66.12 ± 4.83 | 25.87 ± 3.45 / 25.96 ± 5.12 | 33.4 ± 6.8 /  33.3 ± 9.45 | OB/OW | AT | 60%–70% HRmax | 44–55 min/d, 3 x/wk, 12 wks | 89% | Sedentary (Daily routine) | Adiponectin |
| Lee et al., 2012 | Korea | 8 / 8 | 54.75 ± 2.76 / 54.25 ± 2.91 | 25.13 ± 1.63 / 25.19 ± 1.71 | 36.14 ± 3.10 / 36.65 ± 1.85 | OB/OW | AT | 60 min/classes | 60 min/d, 3 x/wk, 16 wks | NR | No exercise | Adiponectin |
| Park et al., 2015 | Korea | 10 / 10 | 57.20 ± 2.57 / 57.20 ± 1.69 | 26.02 ± 1.55 / 26.80 ± 1.09 | 34.32 ± 2.79 / 34.63 ± 1.60 | OB/OW | CT | wks 1–6: 60% 1RM; wks 7–12: 70% 1RM; 40%–70% THR | 30 min/d, 3 x/wk, 12 wks | > 90% | No exercise | TNF-α |
| Phillips et al., 2012 | USA | 11 / 12 | 64.8 ± 2.4 / 66.4 ± 2.8 | 32.2 ± 3.3 /  33.7 ± 4.4 | 35.1 ± 2.7 /  36.4 ± 3.3 | OB/OW | RT | 3 sets of 10 rep 8 RM | 75 min/d, 3 x/wk, 12 wks | 90% | Non-exercise | Adiponectin, CRP, TNF-α |
| Rezende et al., 2016 | Brazil | 19 / 21 | 56.2 ± 7.8 / 54.5 ± 8.9 | 34.1 ± 4.4 /  32 ± 5 | – | OB | AT | from VAT up to 10% below RCP | 50 min/d, 2 x/wk, 20 wks | 85% | Non-exercise | TNF-α |
| Rogers et al., 2014 | USA | 20 / 22 | 55.2 ± 9.1 / 57.2 ± 5.5 | 29.8 ± 4.8 /  32.6 ± 6.6 | – | OB | CT | 48%–52% HRR, 2 sets of 15 rep | 4 x/wk, 3 mo | 87% | Wait-list | IL-6, TNF-α |
| Ryan et al., 2014 | USA | 37 / 40 | 60 ± 1 /  60 ± 1 | 32 ± 1 /  33 ± 1 | 47.1 ± 0.9 /  47.8 ± 0.6 | OB | AT | 50%–85% HRR | 45 min, 3 x/wk, 6 mo | 96% | Diet Only (Weight Loss) | CRP |
| Saeidi et al., 2019 | Iran | 12 / 12 | 58 ± 5 /  56 ± 5 | 27.2 ± 1.8 /  28.2 ± 1.9 | – | OB/OW | RT | 55% 1RM, 2 sets , 12 rep | 3 x/wk, 8 wks | 100% (Supervised) | Placebo / No training | Adiponectin |
| Silverman et al., 2009 | USA | 46 / 40 | 60 ± 5 /  58 ± 5 | 32.1 ± 4.2 /  32.6 ± 4.6 | – | OB | AT | 50%–75% HRR | 3 x/wk, 6 mo | 88% | Diet Only (Weight Loss) | IL-6, TNF-α |
| Son et al., 2023 | Korea | 14 / 12 | 70.2 ± 1.21 / 69.9 ± 1.14 | 26.04 ± 1.94 / 26.06 ± 1.37 | 37.33 ± 3.37 / 37.0 ± 1.57 | OB/OW | AT | 64%–76% HRmax | 110–130 min, 3–5 x/wk, 12 wks | > 90% | No exercise | CRP, IL-6, TNF-α |
| Tomeleri et al., 2016 | Brazil | 19 / 19 | 66.8 ± 3.2 / 69.5 ± 4.7 | 27.8 ± 4.5 /  27.1 ± 3.8 | 42.6 ± 5.7 /  39.5 ± 6.4 | OB/OW | RT | 3 sets of 10–15 RM | 45–50 min/d, 3 x/wk, 8 wks | > 85% | No exercise | CRP, IL-6, TNF-α |
| Tomeleri CM, 2018 | Brazil | 22 / 23 | 72.1 ± 6.3 / 68.8 ± 4.9 | 26.6 ± 3.1 /  27.3 ± 4.2 | 41.1 ± 4.8 /  41.5 ± 6.1 | OB/OW | RT | 3 sets of 10–15 RM | 3 x/wk, 12 wks | > 85% | No exercise | CRP, IL-6, TNF-α |
| Urzi et al., 2019 | Slovenia | 11 / 9 | 84.4 ± 7.7 / 88.9 ± 5.3 | 28.0 ± 5.5 /  29.1 ± 5.1 | – | OB/OW | RT | 12–14 Borg Rate | 50 min/d, 3 x/wk, 12 wks | 88% | Health Education / Usual Care | CRP |
| Vasconcelos et al., 2020 | Brazil | 16 / 11 | 64.88 ± 3.03 / 65.91 ± 5.79 | 29.62 ± 4.99 / 31.77 ± 5.75 | – | OB/OW | CT | 6–7 OMNI–GSE | 45 min, 3 x/wk, 24 wks | > 85% | No exercise | IL-6, TNF-α |
| Wang et al., 2015 | USA | 48 / 22 | 58.4 ± 5.2 / 58.5 ± 6.1 | 32.9 ± 3.7 /  28.7 ± 3.5 | 42.2 ± 2.9 /  41.7 ± 3.9 | OB/OW | AT | 45%–75% HRR | 3 x/wk, 5 mo | 93% | Diet Only (Weight Loss) | Adiponectin |
| Winters-Stone et al., 2017 | USA | 109 / 106 | 59.8 ± 11.4 / 59.3 ± 11.6 | 27.9 ± 5.5 /  28.5 ± 5.3 | – | OB/OW | RT | 60%–80% 1RM | 105 min, 2 x/wk, 12 mo | 81% (Pooled) | Usual Care / Stretching | Adiponectin, CRP, IL-6, TNF-α |

Note: Data are presented as mean ± standard deviation (SD) or range (min–max) unless otherwise indicated. Values separated by a slash (/) represent Experimental Group / Control Group, respectively. Abbreviations: 1RM, one-repetition maximum; ACSM, American College of Sports Medicine; AT, aerobic training; BMI, body mass index; CRP, C-reactive protein; CT, concurrent training (or combined training); Ctrl, control group; d, day; Exp, experimental group; HIIT, high-intensity interval training; HRmax, maximal heart rate; HRR, heart rate reserve; IL-6, interleukin-6; MET, metabolic equivalent of task; min, minute; mo, month; OB, obese; OMNI-GSE, OMNI Global Session Exertion scale; OMNI-RES, OMNI Resistance Exercise Scale; OW, overweight; Post, postmenopausal; Pre, premenopausal; RCP, respiratory compensation point; RM, repetition maximum; RPE, rating of perceived exertion; RT, resistance training; THR, target heart rate; TNF-α, tumor necrosis factor-α; VAT, ventilatory anaerobic threshold; VO₂max/peak, maximal/peak oxygen uptake; wk, week.

**Figure S1.** Summary of the risk of bias assessment


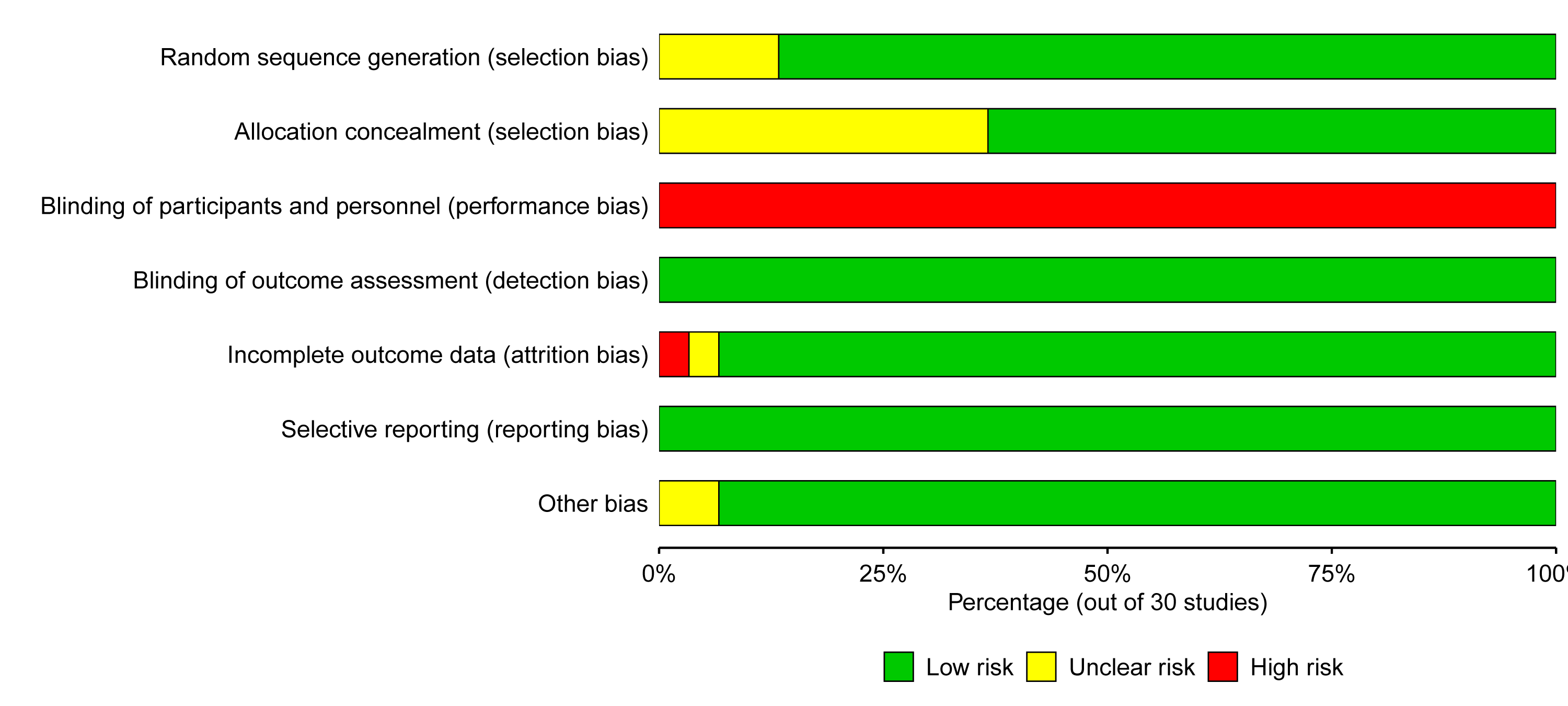


**Figure S2.Traffic light plot of study-level risk of bias assessment.** Green circles (+) indicate a low risk of bias; yellow circles (?) indicate an unclear risk of bias; red circles (-) indicate a high risk of bias. The domains evaluated according to the Cochrane risk-of-bias tool are: D1, Random sequence generation (selection bias); D2, Allocation concealment (selection bias); D3, Blinding of participants and personnel (performance bias); D4, Blinding of outcome assessment (detection bias); D5, Incomplete outcome data (attrition bias); D6, Selective reporting (reporting bias); and D7, Other bias.


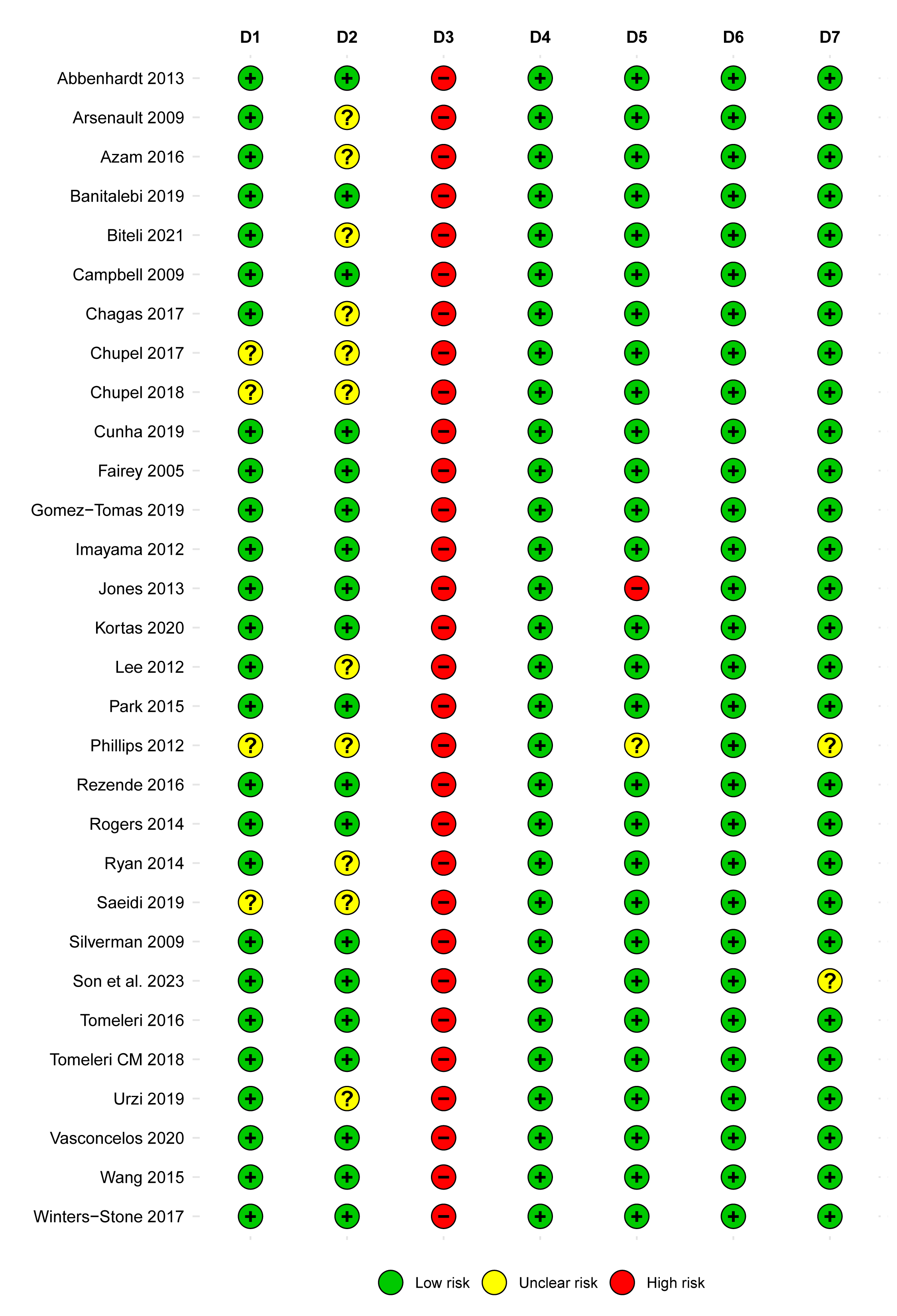


**Figure S3.** Leave-one-out sensitivity analysis for the inflammatory markers and adiponectin.

**
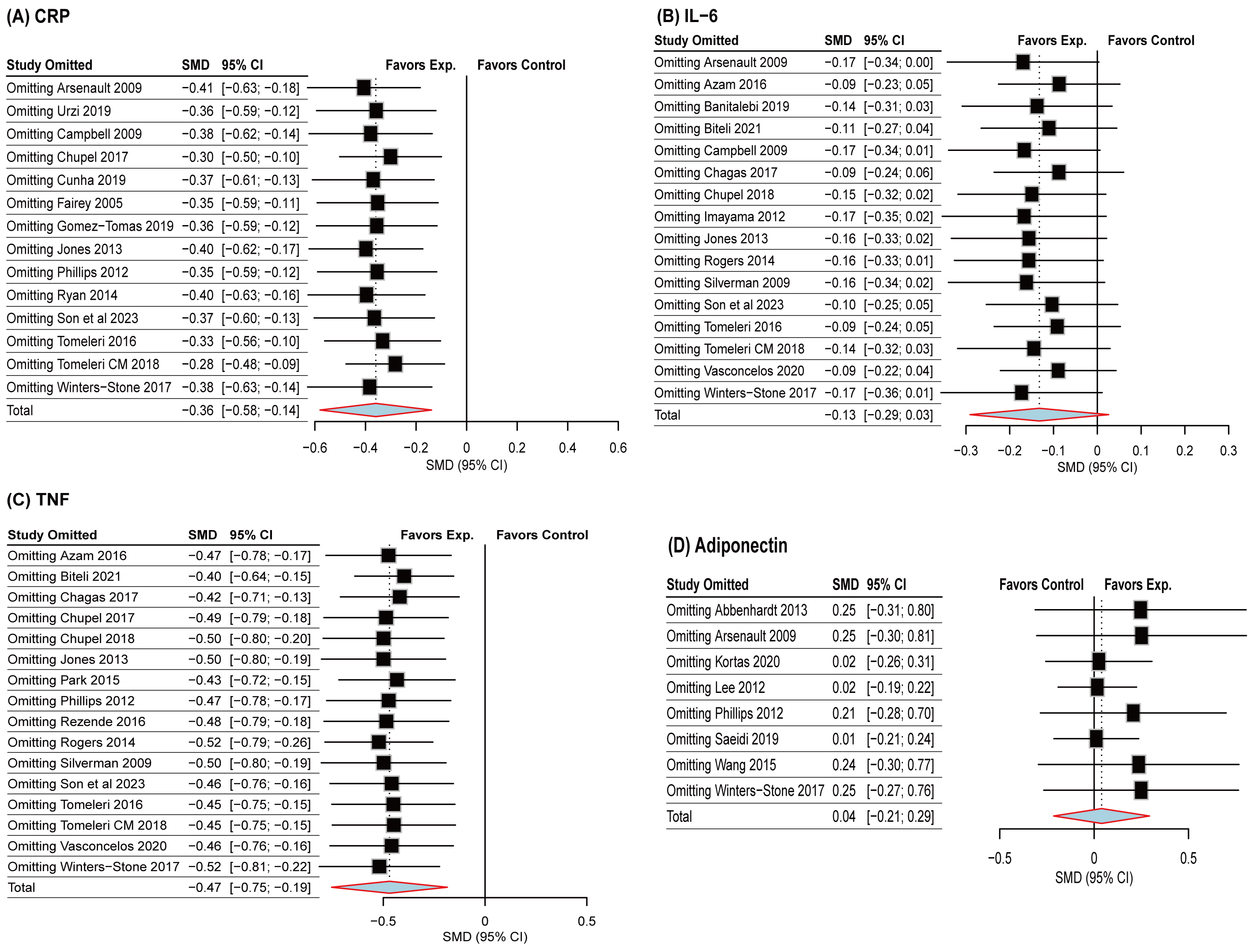
**

**Figure S4. Dose-response meta-regression analysis of total exercise volume.**

**
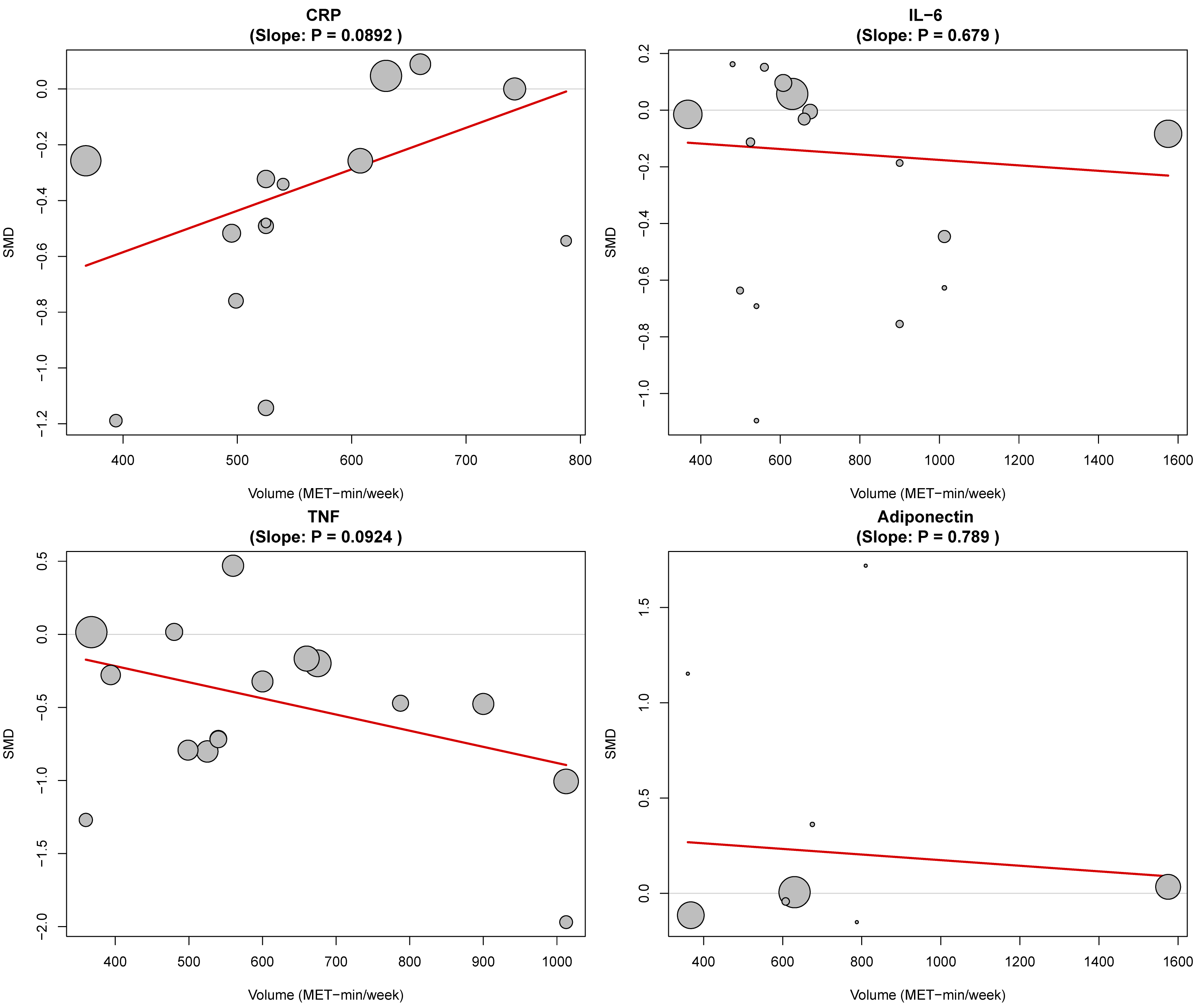
**

**Figure S5.** Dose-response meta-regression analysis of intervention duration.


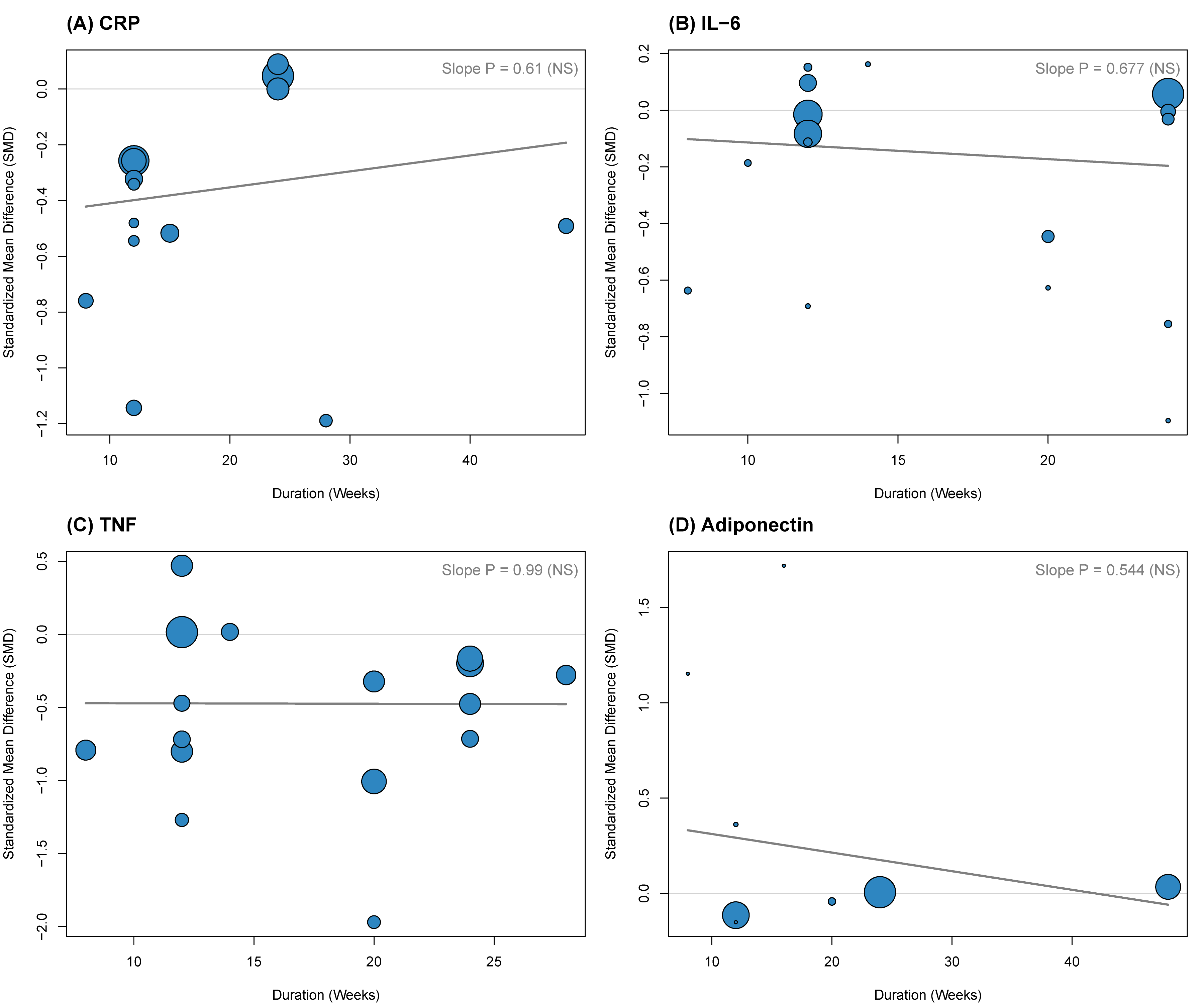


**Figure S6.** Dose-response meta-regression analysis of exercise intensity.


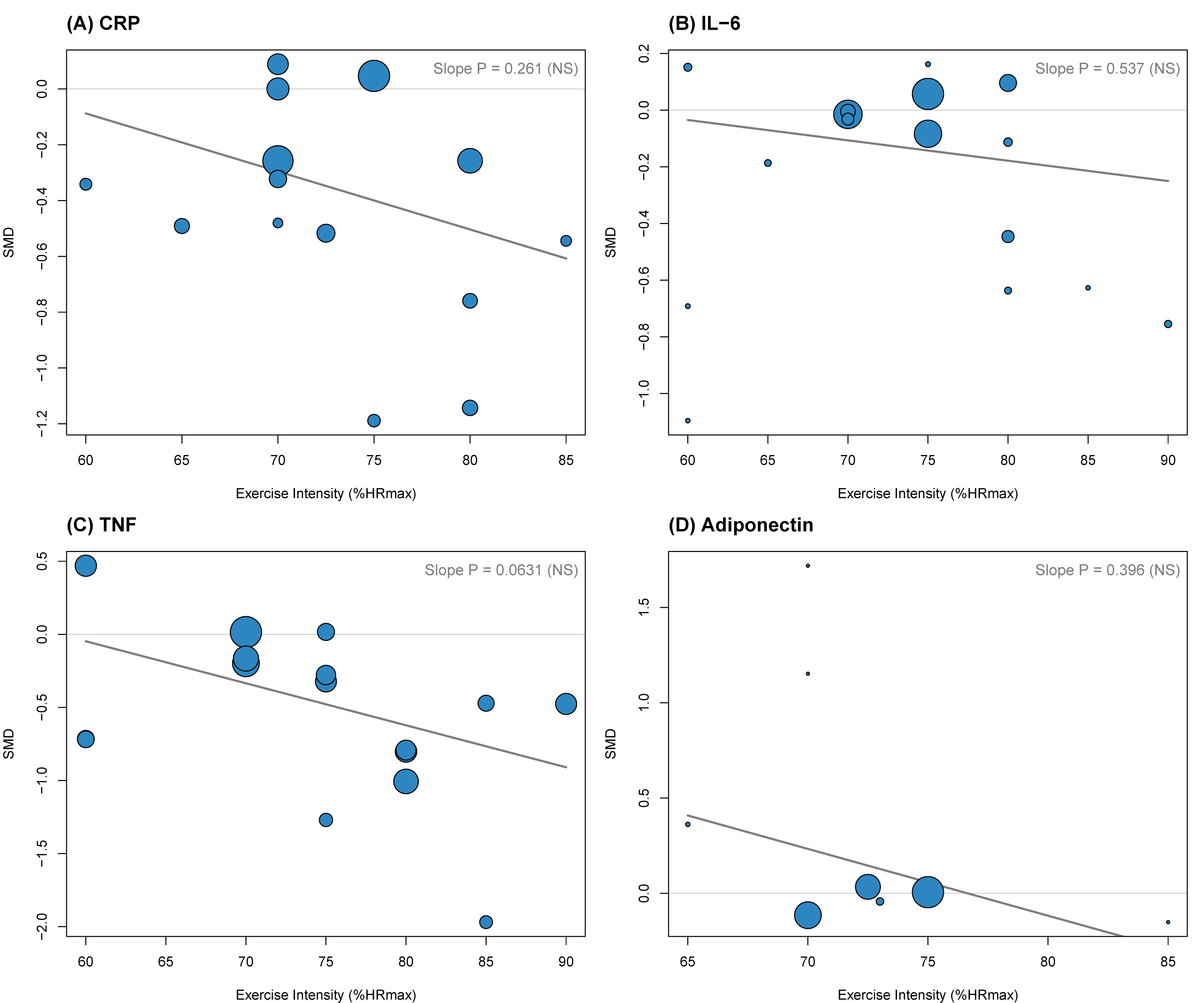


**Table S4.** GRADE certainty of evidence for the primary outcomes

| Markers (Outcomes) | Comparison | Study Limitations | Inconsistency | Indirectness | Imprecision | Publication Bias | Certainty of Evidence (GRADE) |
| --- | --- | --- | --- | --- | --- | --- | --- |
| CRP | Exercise vs. Control | Not serious (a) | Serious (b) | Not serious | Not serious (c) | Serious (d) | LOW |
| TNF-alpha | Exercise vs. Control | Not serious (a) | Serious (b) | Not serious | Not serious (c) | Serious (d) | LOW |
| IL-6 | Exercise vs. Control | Not serious (a) | Serious (b) | Not serious | Serious (e) | Serious (d) | VERY LOW |
| Adiponectin | Exercise vs. Control | Not serious (a) | Serious (b) | Not serious | Serious (e) | Serious (d) | VERY LOW |

**General Note:** All outcomes start at a "High" certainty of evidence because the synthesized data are derived entirely from Randomized Controlled Trials (RCTs).

(a) Study limitations (Risk of Bias): Not downgraded. Although most exercise trials inherently lack blinding of participants and personnel (performance bias), the selected outcomes are objective physiological biomarkers (blood assays). These objective measures are highly unlikely to be influenced by a lack of blinding, thus maintaining the integrity of the results.

(b) Inconsistency: Downgraded one level (-1). There was moderate-to-high statistical heterogeneity observed across the analyses (I-squared range: 32.6% to 63.9%), which is likely attributable to the clinical diversity in exercise modalities, intensities, and total volume across the included trials.

(c) Imprecision (CRP and TNF-alpha): Not downgraded. The 95% confidence intervals (CIs) for both CRP (95% CI: -0.58 to -0.14) and TNF-alpha (95% CI: -0.75 to -0.19) are reasonably narrow and do not cross the line of no effect (SMD = 0), confirming a robust and clinically meaningful direction of effect.

(d) Publication bias: Downgraded one level (-1). Visual inspection of the contour-enhanced funnel plots and formal Egger's linear regression tests indicated statistically significant asymmetry across all four biomarkers (CRP p = 0.009; TNF-alpha p = 0.016; IL-6 p = 0.008; Adiponectin p = 0.040). This strongly suggests the presence of small-study effects or potential publication bias.

(e) Imprecision (IL-6 and Adiponectin): Downgraded one level (-1). The 95% confidence intervals for IL-6 (95% CI: -0.29 to 0.03) and Adiponectin (95% CI: -0.21 to 0.29) are wide and cross the line of no effect (SMD = 0), indicating statistical uncertainty regarding the true intervention effect.

**Table S5.** PRISMA meta-analysis checklist

| **Section/Topic** | **Item** | **Checklist Item** | **Reported in Manuscript** |
| --- | --- | --- | --- |
| **TITLE** |  |  |  |
| Title | 1 | Identify the report as a systematic review, meta-analysis, or both. | Manuscript Title (Title includes "A Dose-Response Meta-Analysis") |
| ABSTRACT |  |  |  |
| Abstract | 2 | See the PRISMA 2020 for Abstracts checklist. | Abstract (Structured: Objective, Methods, Results, Conclusion) |
| **INTRODUCTION** |  |  |  |
| Rationale | 3 | Describe the rationale for the review in the context of existing knowledge. | 1. Introduction |
| Objectives | 4 | Provide an explicit statement of the objective(s) or question(s) the review addresses. | 1. Introduction (Last paragraph: "To address these complexities...") |
| **METHODS** |  |  |  |
| Eligibility criteria | 5 | Specify the inclusion and exclusion criteria (PICOS) and the report characteristics. | 2.1 Literature Search Strategy & 2.3 Eligibility Criteria |
| Information sources | 6 | Specify all databases, registers, websites, and other sources searched. | 2.1 Literature Search Strategy |
| Search strategy | 7 | Present the full search strategies for all databases and registers. | 2.1 Literature Search Strategy & Supplementary Table S1 |
| Selection process | 8 | Specify the methods used to decide whether a study met the inclusion criteria. | 2.4 Study Selection and Data Extraction |
| Data collection process | 9 | Specify the methods used to collect data from reports. | 2.4 Study Selection and Data Extraction |
| Data items | 10 | List and define all outcomes for which data were sought. | 2.3 Eligibility Criteria & 2.4 Study Selection and Data Extraction |
| Study risk of bias assessment | 11 | Specify the methods used to assess risk of bias in the included studies. | 2.5 Risk of Bias and Methodological Quality Assessment |
| Effect measures | 12 | Specify the effect measure(s) used in the synthesis. | 2.6 Data Synthesis and Statistical Analysis |
| Synthesis methods | 13 | Describe the methods used to decide which studies were eligible for each synthesis. | 2.6 Data Synthesis and Statistical Analysis |
| Reporting bias assessment | 14 | Describe any methods used to assess risk of reporting bias. | 2.6 Data Synthesis and Statistical Analysis |
| Certainty assessment | 15 | Describe any methods used to assess certainty in the body of evidence. | Supplementary Table S4 (GRADE approach) |
| **RESULTS** |  |  |  |
| Study selection | 16 | Describe the results of the search and selection process (flow diagram). | 3.1 Characteristics of Included Studies & Figure 1 |
| Study characteristics | 17 | Cite each included study and present its characteristics. | 3.1 Characteristics of Included Studies & Supplementary Table S3 |
| Risk of bias in studies | 18 | Present assessments of risk of bias for each included study. | Supplementary Figure S1 & Supplementary Figure S2 |
| Results of individual studies | 19 | For all outcomes, present the summary statistics for each intervention group. | Figure 2 (Forest plots) |
| Results of syntheses | 20 | Summarize the results of the syntheses and present the results of meta-analyses. | 3.2 Effects of Exercise on Inflammatory Markers and Adiponectin |
| Dose-response analysis | 20a* | Present meta-regression results for continuous moderators. | 3.4 Dose-Response Analysis & Supplementary Figures S4–S6 |
| Reporting biases | 21 | Present assessments of risk of reporting bias. | 3.5 Publication Bias & Figure 3 (Funnel plots) |
| Certainty of evidence | 22 | Present assessments of certainty in the body of evidence for each outcome. | Supplementary Table S4 |
| **DISCUSSION** |  |  |  |
| Discussion | 23 | Provide a general interpretation of the results, discuss limitations, and outline implications. | 4. Discussion (4.1, 4.2, 4.3) & 4.4 Strengths and Limitations & 5. Conclusion |
| **OTHER INFORMATION** |  |  |  |
| Registration and protocol | 24 | Provide registration information for the review. | 2. Methods (PROSPERO: CRD420261288134) |
| Support | 25 | Describe sources of financial or non-financial support. | Funding |
| Competing interests | 26 | Declare any competing interests of review authors. | Conflict of Interest |
| Availability of data, code | 27 | Report which of the following are publicly available. | Data Availability Statement |

**References:**

Abbenhardt, C., McTiernan, A., Alfano, C.M., Wener, M.H., Campbell, K.L., Duggan, C., Foster-Schubert, K.E., Kong, A., Toriola, A.T., Potter, J.D., Mason, C., Xiao, L., Blackburn, G.L., Bain, C., and Ulrich, C.M. (2013). Effects of individual and combined dietary weight loss and exercise interventions in postmenopausal women on adiponectin and leptin levels. *J Intern Med* 274**,** 163-175.

Arsenault, B.J., Côté, M., Cartier, A., Lemieux, I., Després, J.P., Ross, R., Earnest, C.P., Blair, S.N., and Church, T.S. (2009). Effect of exercise training on cardiometabolic risk markers among sedentary, but metabolically healthy overweight or obese post-menopausal women with elevated blood pressure. *Atherosclerosis* 207**,** 530-533.

Azam, Abdollahpour, Nasim, Khosravi, Zohreh, Eskandari, Shahpar, and Haghighat (2016). Effect of Six Months of Aerobic Exercise on Plasma Interleukin-6 and Tumor Necrosis Factor-Alpha as Breast Cancer Risk Factors in Postmenopausal Women: A Randomized Controlled Trial. *Iranian Red Crescent Medical Journal*.

Banitalebi, E., Kazemi, A., Faramarzi, M., Nasiri, S., and Haghighi, M.M. (2019). Effects of sprint interval or combined aerobic and resistance training on myokines in overweight women with type 2 diabetes: A randomized controlled trial. *Life Sci* 217**,** 101-109.

Biteli, P., Barbalho, S.M., Detregiachi, C.R.P., Dos Santos Haber, J.F., and Chagas, E.F.B. (2021). Dyslipidemia influences the effect of physical exercise on inflammatory markers on obese women in post-menopause: A randomized clinical trial. *Exp Gerontol* 150**,** 111355.

Campbell, P.T., Campbell, K.L., Wener, M.H., Wood, B.L., Potter, J.D., McTiernan, A., and Ulrich, C.M. (2009). A yearlong exercise intervention decreases CRP among obese postmenopausal women. *Med Sci Sports Exerc* 41**,** 1533-1539.

Chagas, E.F.B., Bonfim, M.R., Turi, B.C., Brondino, N.C.M., and Monteiro, H.L. (2017). Effect of Moderate-Intensity Exercise on Inflammatory Markers Among Postmenopausal Women. *J Phys Act Health* 14**,** 479-485.

Chupel, M.U., Direito, F., Furtado, G.E., Minuzzi, L.G., Pedrosa, F.M., Colado, J.C., Ferreira, J.P., Filaire, E., and Teixeira, A.M. (2017). Strength Training Decreases Inflammation and Increases Cognition and Physical Fitness in Older Women with Cognitive Impairment. *Front Physiol* 8**,** 377.

Chupel, M.U., Minuzzi, L.G., Furtado, G., Santos, M.L., Hogervorst, E., Filaire, E., and Teixeira, A.M. (2018). Exercise and taurine in inflammation, cognition, and peripheral markers of blood-brain barrier integrity in older women. *Appl Physiol Nutr Metab* 43**,** 733-741.

Cunha, P.M., Ribeiro, A.S., Nunes, J.P., Tomeleri, C.M., Nascimento, M.A., Moraes, G.K., Sugihara, P.J., Barbosa, D.S., Venturini, D., and Cyrino, E.S. (2019). Resistance training performed with single-set is sufficient to reduce cardiovascular risk factors in untrained older women: The randomized clinical trial. Active Aging Longitudinal Study. *Arch Gerontol Geriatr* 81**,** 171-175.

Fairey, A.S., Courneya, K.S., Field, C.J., Bell, G.J., Jones, L.W., Martin, B.S., and Mackey, J.R. (2005). Effect of exercise training on C-reactive protein in postmenopausal breast cancer survivors: a randomized controlled trial. *Brain Behav Immun* 19**,** 381-388.

Gomez-Tomas, C., Chulvi-Medrano, I., Josecarrasco, J.J., and Alakhdar, Y. (2019). Effect of a 1-year elastic band resistance exercise program on cardiovascular risk profile in postmenopausal women. *Menopause (New York, N.Y.)* 2018年25卷9期**,** 1004-1010页.

Imayama, I., Ulrich, C.M., Alfano, C.M., Wang, C., Xiao, L., Wener, M.H., Campbell, K.L., Duggan, C., Foster-Schubert, K.E., Kong, A., Mason, C.E., Wang, C.Y., Blackburn, G.L., Bain, C.E., Thompson, H.J., and McTiernan, A. (2012). Effects of a caloric restriction weight loss diet and exercise on inflammatory biomarkers in overweight/obese postmenopausal women: a randomized controlled trial. *Cancer Res* 72**,** 2314-2326.

Jones, S.B., Thomas, G.A., Hesselsweet, S.D., Alvarez-Reeves, M., Yu, H., and Irwin, M.L. (2013). Effect of exercise on markers of inflammation in breast cancer survivors: the Yale exercise and survivorship study. *Cancer Prev Res (Phila)* 6**,** 109-118.

Kortas, J., Ziemann, E., Juszczak, D., Micielska, K., Kozłowska, M., Prusik, K., Prusik, K., and Antosiewicz, J. (2020). Iron Status in Elderly Women Impacts Myostatin, Adiponectin and Osteocalcin Levels Induced by Nordic Walking Training. *Nutrients* 12.

Lee, J.A., Kim, J.W., and Kim, D.Y. (2012). Effects of yoga exercise on serum adiponectin and metabolic syndrome factors in obese postmenopausal women. *Menopause* 19**,** 296-301.

Park, S.M., Kwak, Y.S., and Ji, J.G. (2015). The Effects of Combined Exercise on Health-Related Fitness, Endotoxin, and Immune Function of Postmenopausal Women with Abdominal Obesity. *J Immunol Res* 2015**,** 830567.

Phillips, M.D., Patrizi, R.M., Cheek, D.J., Wooten, J.S., Barbee, J.J., and Mitchell, J.B. (2012). Resistance training reduces subclinical inflammation in obese, postmenopausal women. *Med Sci Sports Exerc* 44**,** 2099-2110.

Rezende, R.E., Duarte, S.M., Stefano, J.T., Roschel, H., Gualano, B., de Sá Pinto, A.L., Vezozzo, D.C., Carrilho, F.J., and Oliveira, C.P. (2016). Randomized clinical trial: benefits of aerobic physical activity for 24 weeks in postmenopausal women with nonalcoholic fatty liver disease. *Menopause* 23**,** 876-883.

Rogers, L.Q., Vicari, S., Trammell, R., Hopkins-Price, P., Fogleman, A., Spenner, A., Rao, K., Courneya, K.S., Hoelzer, K.S., Robbs, R., and Verhulst, S. (2014). Biobehavioral factors mediate exercise effects on fatigue in breast cancer survivors. *Med Sci Sports Exerc* 46**,** 1077-1088.

Ryan, A.S., Ge, S., Blumenthal, J.B., Serra, M.C., Prior, S.J., and Goldberg, A.P. (2014). Aerobic exercise and weight loss reduce vascular markers of inflammation and improve insulin sensitivity in obese women. *J Am Geriatr Soc* 62**,** 607-614.

Saeidi, A., Jabbour, G., Ahmadian, M., Abbassi-Daloii, A., Malekian, F., Hackney, A.C., Saedmocheshi, S., Basati, G., Ben Abderrahman, A., and Zouhal, H. (2019). Independent and Combined Effects of Antioxidant Supplementation and Circuit Resistance Training on Selected Adipokines in Postmenopausal Women. *Front Physiol* 10**,** 484.

Silverman, N.E., Nicklas, B.J., and Ryan, A.S. (2009). Addition of aerobic exercise to a weight loss program increases BMD, with an associated reduction in inflammation in overweight postmenopausal women. *Calcif Tissue Int* 84**,** 257-265.

Son, W.H., Park, H.T., Jeon, B.H., and Ha, M.S. (2023). Moderate intensity walking exercises reduce the body mass index and vascular inflammatory factors in postmenopausal women with obesity: a randomized controlled trial. *Sci Rep* 13**,** 20172.

Tomeleri, C.M., Ribeiro, A.S., Souza, M.F., Schiavoni, D., Schoenfeld, B.J., Venturini, D., Barbosa, D.S., Landucci, K., Sardinha, L.B., and Cyrino, E.S. (2016). Resistance training improves inflammatory level, lipid and glycemic profiles in obese older women: A randomized controlled trial. *Exp Gerontol* 84**,** 80-87.

Tomeleri CM, S.M., Burini RC, et al. (2018). Resistance training reduces metabolic syndrome and inflammatory markers in older women: A randomized controlled trial. *Journal of Diabetes* 10.

Urzi, F., Marusic, U., Ličen, S., and Buzan, E. (2019). Effects of Elastic Resistance Training on Functional Performance and Myokines in Older Women—A Randomized Controlled Trial. *Journal of the American Medical Directors Association*.

Vasconcelos, A.B.S., Resende-Neto, A.G.D., Nogueira, A.C., Santos, J.C.A., and Silva-Grigoletto, M.E.D. (2020). Functional and traditional training improve muscle power and reduce proinflammatory cytokines in older women: A randomized controlled trial. *Experimental Gerontology* 135**,** 110920.

Wang, X., You, T., Murphy, K., Lyles, M.F., and Nicklas, B.J. (2015). Addition of Exercise Increases Plasma Adiponectin and Release from Adipose Tissue. *Med Sci Sports Exerc* 47**,** 2450-2455.

Winters-Stone, K.M., Wood, L.J., Stoyles, S., and Dieckmann, N.F. (2017). The Effects of Resistance Exercise on Biomarkers of Breast Cancer Prognosis: A Pooled Analysis of Three Randomized Trials. *Cancer Epidemiology Biomarkers & Prevention*.
